# Neuropilin-2 functions as a coinhibitory receptor to regulate antigen-induced inflammation and allograft rejection

**DOI:** 10.1172/JCI172218

**Published:** 2025-07-01

**Authors:** Johannes Wedel, Nora Kochupurakkal, Sek Won Kong, Sayantan Bose, Ji-Won Lee, Madeline Maslyar, Bayan Alsairafi, Kayla MacLeod, Kaifeng Liu, Hengcheng Zhang, Masaki Komatsu, Hironao Nakayama, Diane R. Bielenberg, David M. Briscoe

**Affiliations:** 1 Transplant Research Program and; 2 Division of Nephrology, Department of Pediatrics, Boston Children’s Hospital, Boston, Massachusetts, USA.; 3 Department of Pediatrics, Harvard Medical School, Boston, Massachusetts, USA.; 4 Computational Health Informatics Program, Boston Children’s Hospital, Boston, Massachusetts, USA.; 5 Transplantation Research Center, Renal Division, Department of Medicine, Brigham and Women’s Hospital, Boston, Massachusetts, USA.; 6 Department of Medicine, Harvard Medical School, Boston, Massachusetts, USA.; 7 Vascular Biology Program, Boston Children’s Hospital, Boston, Massachusetts, USA.; 8 Department of Surgery, Harvard Medical School, Boston, Massachusetts, USA.

**Keywords:** Immunology, Transplantation, Cellular immune response, Organ transplantation, T cells

## Abstract

Coinhibitory receptors function as central modulators of the immune response to resolve T effector activation and/or to sustain immune homeostasis. Here, using humanized SCID mice, we found that neuropilin–2 (NRP2) is inducible on late effector and exhausted subsets of human CD4^+^ T cells and that it is coexpressed with established coinhibitory molecules including PD-1, CTLA4, TIGIT, LAG3, and TIM3. In murine models, we also found that NRP2 is expressed on effector memory CD4^+^ T cells with an exhausted phenotype and that it functions as a key coinhibitory molecule. Knockout (KO) of NRP2 resulted in hyperactive CD4^+^ T cell responses and enhanced inflammation in delayed-type hypersensitivity and transplantation models. After cardiac transplantation, allograft rejection and graft failure were accelerated in global as well as CD4^+^ T cell–specific KO recipients, and enhanced alloimmunity was dependent on NRP2 expression on CD4^+^ T effectors but not on CD4^+^Foxp3^+^ Tregs. Also, KO Tregs were found to be as efficient as WT cells in the suppression of effector responses in vitro and in vivo. These collective findings identify NRP2 as a potentially novel coinhibitory receptor and demonstrate that its expression on CD4^+^ T effector cells is of great functional importance in immunity.

## Abstract 2

Knockout of Neuropilin-2 within CD4^+^ T effector cells results in enhanced antigen-specific and cell-mediated inflammatory responses, and accelerated rejection of cardiac allografts.

## Graphical abstract

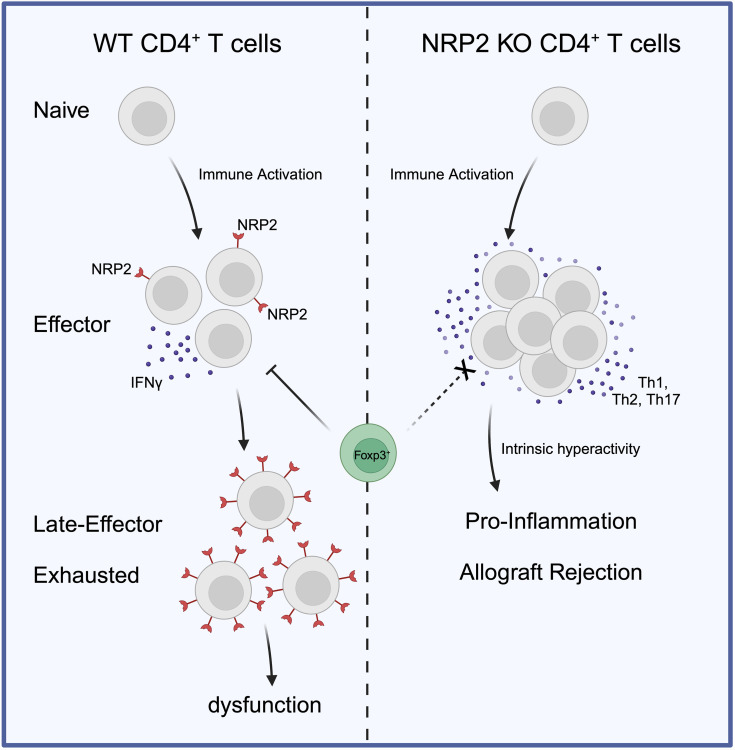

## Introduction

T cell activation is intrinsically regulated and self-limited to prevent chronic inflammation and autoimmunity. One such regulatory mechanism is called T cell exhaustion (Texh cells, also called T cell dysfunction), characterized by reduced T effector function including loss of cytokine production and reduced proliferation rates (1). Differentiation into Texh is reported to be partly dependent on the induced expression of coinhibitory receptors including PD-1, CTLA4, TIGIT, LAG3, TIM-3, and BTLA (1–4). These coinhibitory receptors collectively function to suppress ongoing cell-mediated immune activation, which ultimately serves to resolve the inflammatory response. Augmentation of Texh has multiple regulatory effects on the immune response, including an established effect to limit alloimmunity and prolong graft survival after transplantation (5). In contrast, blockade of individual coinhibitory receptors may reverse Texh, for example, to enhance tumor immune responses (6–12). Thus, understanding the cellular basis for Texh/T cell dysfunction has broad clinical implications (5, 13–15).

The neuropilin (NRP) receptors NRP1 and NRP2 are type I transmembrane glycoproteins that were initially identified as chemorepulsive axonal guidance receptors (16–19). These receptors bind multiple ligands, including class III semaphorins (SEMA3), VEGF-A and VEGF-C, and TGF-β (17–24). They are expressed by multiple cell types and function in a broad range of biological processes, including cytoskeletal stability, migration, angiogenesis, and cell growth (25–29). NRP1 also functions to regulate cell-mediated immune responses via its expression on both T cells and antigen presenting cells (APCs) (30, 31). Indeed, a dominant biological effect of NRP1 relates to its expression on CD4^+^ Tregs where it augments immunoregulation and lineage stability (32–36). Deletion of NRP1 on CD4^+^ T cells is reported to increase disease severity in models of experimental autoimmune encephalitis (37) and colitis (34). Also, NRP1 is reported to regulate CD8^+^ T cell memory responses in association with antitumor immunity (38, 39).

In this study, we identified NRP2 on subsets of human and mouse immune cells, and we showed that it is inducible on antigen-activated CD4^+^ T cells, most notably on late effector and Texh subsets. Using NRP2 knockout (KO) mice, we found that NRP2 functions to regulate CD4^+^ T cell activation in vitro as well as in cell-mediated immune responses after vaccination and transplantation in vivo. We also found that KO of NRP2 on CD4^+^ effector T cells (Teffs), but not on CD4^+^Foxp3^+^ Tregs, was associated with accelerated rejection and enhanced alloimmunity after cardiac transplantation. Furthermore, in a skin transplantation model, we found that NRP2 was redundant on Tregs, as deletion did not alter their immune suppressive function. Overall, our findings demonstrated that NRP2 is a coinhibitory receptor on CD4^+^ T cells and that it functions to regulate antigen-activated and effector immune responses, most notably after transplantation.

## Results

### NRP2 is expressed by multiple immune cell subsets and is inducible on subpopulations of CD4^+^ T cells in the course of an immune response.

**Figure 1:**
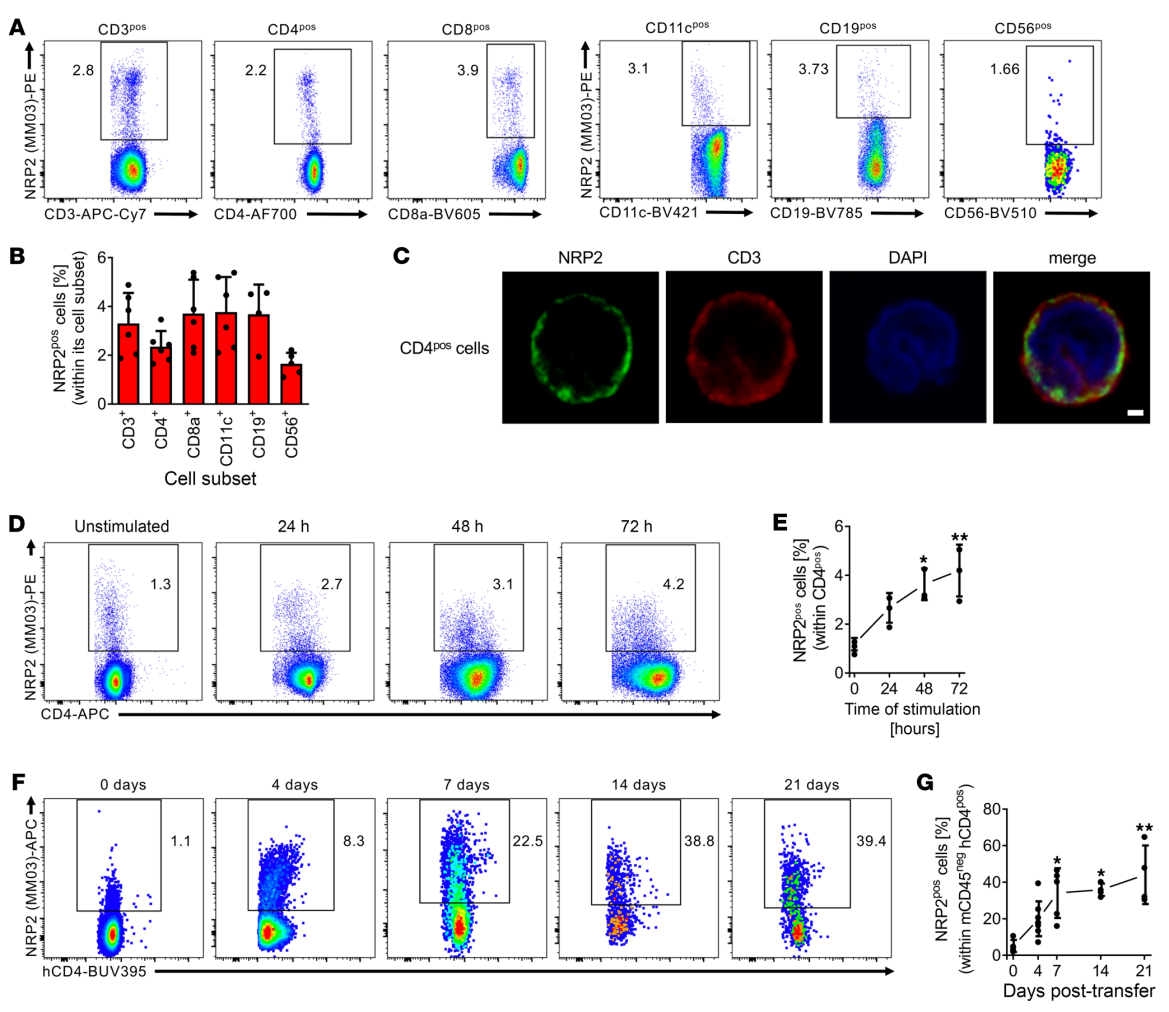
Inducible NRP2 expression on distinct subsets of human CD4^+^ T cells. (**A**) Representative dot plots and (**B**) a summary of 6 independent flow cytometric analyses (mean ± SD) of NRP2 staining of freshly isolated human PBMCs. (**C**) Representative cytospin of negatively isolated human CD4^+^ T cells stained for NRP2 (clone MM03; green) and CD3 (red); imaged by confocal microscopy. Representative images of 4 independent experiments showing an NRP2-expressing CD4^+^ cell at high-power magnification. Scale bar: 1 μm. (**D**) Representative dot plots of NRP2 expression on human CD4^+^ T cells cultured in the presence of phytohemagglutinin (PHA; 3 μg/mL for up to 72 hours) and evaluated by flow cytometry. (**E**) Line graph summarizing 3 independent experiments (data shown as mean ± SD; Friedman’s test with Dunn’s multiple-comparison test, *P < 0.05, **P < 0.01). (**F**) NRP2 expression by flow cytometry on human CD4^+^ T cells within splenocytes of huSCID mice at selected time intervals after humanization. Dot plots are gated on human CD4^+^ cells (murine CD45^neg^). (**G**) Line graph illustrating changes in the expression of NRP2 on CD4^+^ T cells over a 21-day period after humanization of huSCID mice (*n* = 4–8 independent experiments per time point; data shown as mean ± SD; Kruskal-Wallis test with Dunn’s multiple-comparison test, *P < 0.05, **P < 0.01).

We initially profiled NRP2 expression on human PBMCs. As illustrated in Figure 1, A and B, it is expressed on several cell types, including CD4^+^ and CD8^+^ T cells, CD19^+^ B cells, and CD11c^+^ APCs. Moreover, we consistently found a notable expression pattern on a subpopulation of CD4^+^ T cells by flow cytometry (Figure 1A, left panel, and Supplemental Figure 1; supplemental material available online with this article; https://doi.org/10.1172/JCI172218DS1). Expression was prominent on isolated CD4^+^ T cells by immunofluorescence staining and confocal microscopy (Figure 1C). Also, we found that the level of NRP2 expression on distinct subpopulations of CD4^+^ T cells was sustained after mitogen activation (Figure 1, D and E).

We next transferred PBMCs into lymphopenic SCID-beige mice, harvested the spleens, and evaluated expression on transferred cells as a time course for up to 21 days (Figure 1, F and G, and Supplemental Figure 2). By flow cytometry, we found a marked induction in NRP2 expression on CD4^+^ T cells (Figure 1, F and G) as well as on CD8^+^ T cells, CD11c^+^ APCs, and CD19^+^ B cells (Supplemental Figure 2, D–G). Expression within the CD4^+^ population increased from less than 3% cells at baseline to over 30% cells on day 14 and up to approximately 45% cells over the 21-day time course (Figure 1G). Collectively, these findings indicate that NRP2 is expressed on multiple immune cell lineages but is markedly inducible on distinct subsets of CD4^+^ T cells after activation in vivo.

### Coexpression of NRP2 with multiple coinhibitory receptors on CD4^+^ T cells.

**Figure 2:**
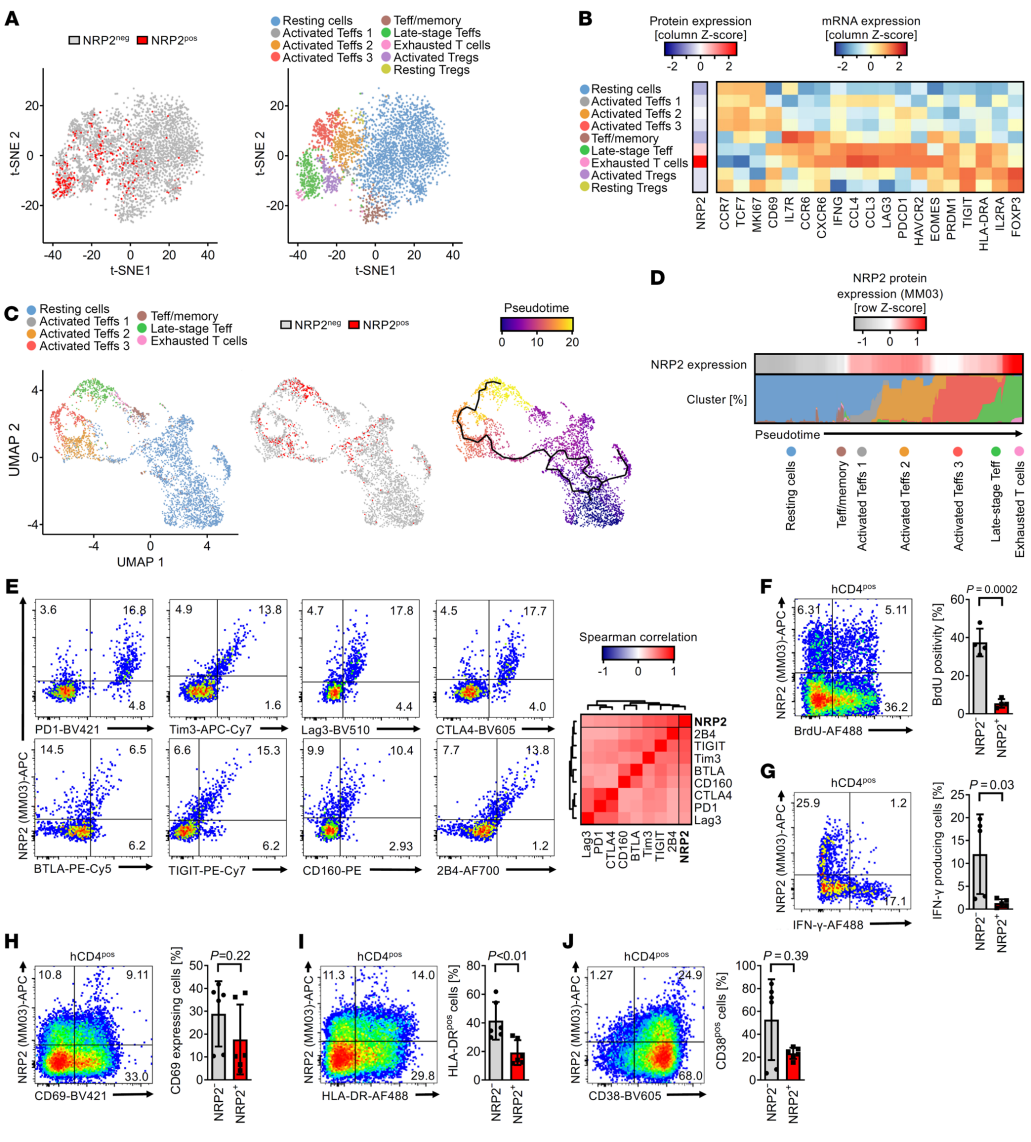
Patterns of expression of NRP2 on late-stage effector and exhausted CD4^+^ T cells. Human CD4^+^ T cells were isolated from the spleens of huSCID mice on day 7 after humanization; the cells were stained with DNA- and fluorochrome-conjugated anti-NRP2 antibodies and subsequently sorted by FACS for proteogenomic analysis using CITE-Seq. (**A**) t-SNE plots depicting NRP2 protein expression (left) and CD4^+^ T cell subset clusters based on transcriptomes (right). (**B**) Heatmap of cluster-defining transcripts for **A**. (**C**) UMAP plots with embedding of Teff populations (excluding Treg clusters) with color coding of each cluster (left), color coding of NRP2 protein expression (middle), and color coding of the calculated pseudotime (right; solid black line represents the pseudotime trajectory). (**D**) Heatmap (top) and stacked density color-coded blot representing the level of NRP2 protein expression and T cell subset distribution over the pseudotime in **C**. (**E**) Representative flow cytometric expression of co-inhibitory molecules on isolated CD4^+^ T cells (left) and a heatmap illustrating the mean Spearman’s rank correlation coefficient between NRP2 and each co-inhibitory receptor in 4 independent experiments (right). (**F**) huSCID mice were pulsed with BrdU on days 4–7 after transfer and spleens were harvested on day 7; the proliferation of human CD4^+^ T cells was assessed by intracellular BrdU staining using flow cytometry. A representative dot plot (left) and a bar graph summary of BrdU positivity in *n* = 4 mice (right) are depicted (data shown as mean ± SD; unpaired *t* test). (**G**) Intracellular IFN-γ staining of human CD4^+^ cells (day 7 after transfer). A representative dot plot (left) and a summary of IFN-γ–producing cells in *n* = 5 mice (right) are depicted (data shown as mean ± SD; Mann-Whitney *U* test). (**H**–**J**) Expression of the activation markers CD69, HLA-DR, and CD38 on human CD4^+^ cells (day 7). Representative dot plots (left) and a summary of expression in *n* = 6 mice (right) are depicted (data shown as mean ± SD; **H** and **I**: unpaired *t* test, **J**: Mann-Whitney *U* test).

To identify the subpopulations of CD4^+^ cells that express NRP2, we performed cellular indexing of transcriptomes and epitopes (CITE) sequencing (scRNA-Seq) on CD4^+^ T cells that were isolated on day 7 after transfer in the huSCID model (Figure 1F and Supplemental Figure 3A). t-distributed stochastic neighbor embedding (t-SNE) dimensional reduction and clustering was performed (Figure 2A), and NRP2 positivity was evaluated using 2 NRP2 antibodies (Supplemental Figure 3B). We found that NRP2 concentrated in 2 clusters belonging to PD-1^+^TIM-3^+^EOMES^neg^ late-stage T effectors and PD-1^+^TIM-3^+^EOMES^pos^ exhausted subsets (Figure 2B and Supplemental Figure 3C). Pseudotime trajectory analysis (Figure 2, C and D) was used to characterize NRP2 expression during differentiation from naive resting CD4^+^ T cells through activated and late-stage Teffs and ultimately within Texh cell subsets (Figure 2C). Heatmap analysis along the pseudotime trajectory indicated that NRP2 expression is initiated during Teff cell activation and peaks during later stages of Teff differentiation in association with exhaustion (Figure 2D). Finally, we performed flow cytometric analysis at multiple time points after transfer of PBMCs in the huSCID mouse and found that NRP2 colocalizes with established coinhibitory molecules, including PD-1, CTLA4, TIGIT, LAG-3, and TIM-3, further confirming that it is expressed on phenotypic Texh subsets (Figure 2E and Supplemental Figure 3, D and E). Consistent with this phenotype, NRP2^pos^ subsets had a significantly lower proliferation rate (as assessed by BrdU incorporation) and produced minimal IFN-γ compared with NRP2^neg^ CD4^+^ T cells (Figure 2, F and G). These findings suggest that NRP2^pos^ cells are both phenotypically and functionally exhausted. Finally, expression of CD69, CD38, and HLA-DR was not associated with NRP2 positivity, indicating that it is not a general marker of activation (Figure 2, H–J). Collectively, these data identified NRP2 on late-stage effector and Texh CD4^+^ T cell subsets.

### NRP2-expressing CD4^+^ T effector cells are enriched with genes associated with T cell dysfunction.

**Figure 3:**
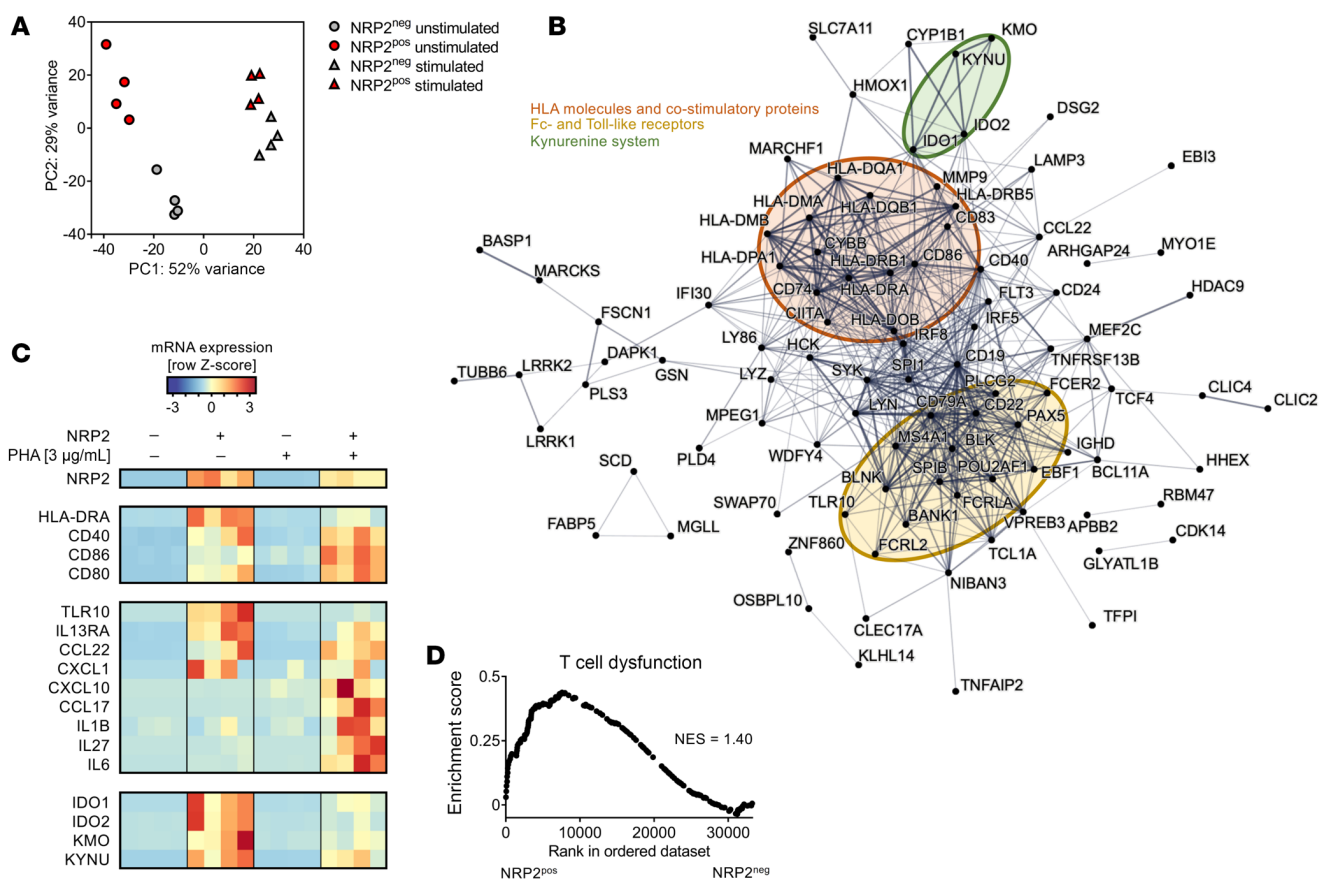
NRP2^pos^ CD4^+^ T cells have a distinct transcriptional profile. Pooled populations of human CD4^+^ T cells were stimulated with PHA (3 μg/mL) for 16 hours in vitro, and NRP2^pos^ and NRP2^neg^ cells were sorted by flow cytometry and subjected to transcriptomic analysis. (**A**) Principal component analysis of unstimulated and stimulated subsets. (**B**) Protein-protein interaction analysis using NRP2 coregulated transcripts (≥2.5 log fold-change and *Padj* < 10^–10^ between unstimulated NRP2^pos^ and NRP2^neg^ cells shown in **A**. Subnetwork nodes are highlighted in color. (**C**) Heatmap depicting transcripts identified in protein interaction network analyses in **B** and additional transcripts that were upregulated in NRP2^pos^ cells after PHA stimulation. (**D**) Enrichment for genes associated with dysfunctional T cells (90) using gene set enrichment analysis. Ranked in order for NRP2^pos^ to NRP2^neg^ cells in unstimulated conditions (NES, normalized enrichment score).

Bulk RNA-Seq of FACS-sorted NRP2^pos^ and NRP2^neg^ CD4^+^ T cells revealed that NRP2^pos^ subsets have a unique transcriptomic signature that differentiates them from NRP2^neg^ cells (Figure 3A and Supplemental Figure 4). Furthermore, this signature was sustained after activation with PHA (Figure 3A). In unstimulated conditions, differences among NRP2^pos^ and NRP2^neg^ cells include subclusters of transcripts for costimulatory molecules, chemokine receptors, and kynurenine system molecules (Figure 3, B and C). In the presence of mitogen, differences also include transcripts for select cytokines including IL-6 (Figure 3C). Additionally, gene set enrichment analyses indicated that unstimulated NRP2^pos^ CD4^+^ T cell subsets expressed transcripts associated with exhaustion/dysfunction (Figure 3D), and this transcriptome was sustained after mitogen activation (data not shown). Finally, computed intrinsic differences in signaling pathway activities were minimal in unstimulated NRP2^pos^ and NRP2^neg^ CD4^+^ subsets (Supplemental Figure 4C), whereas mitogen activation resulted in identifiable effects of NRP2 on pathways that included NF-κB, STAT3, and cell cycle–associated signaling (Supplemental Figure 4C). Collectively, these findings indicate that NRP2^pos^ CD4^+^ T cells possess a unique transcriptome, and thus are likely a distinct subset.

### NRP2 serves as a coinhibitory receptor and regulates CD4^+^ T effector function.

**Figure 4:**
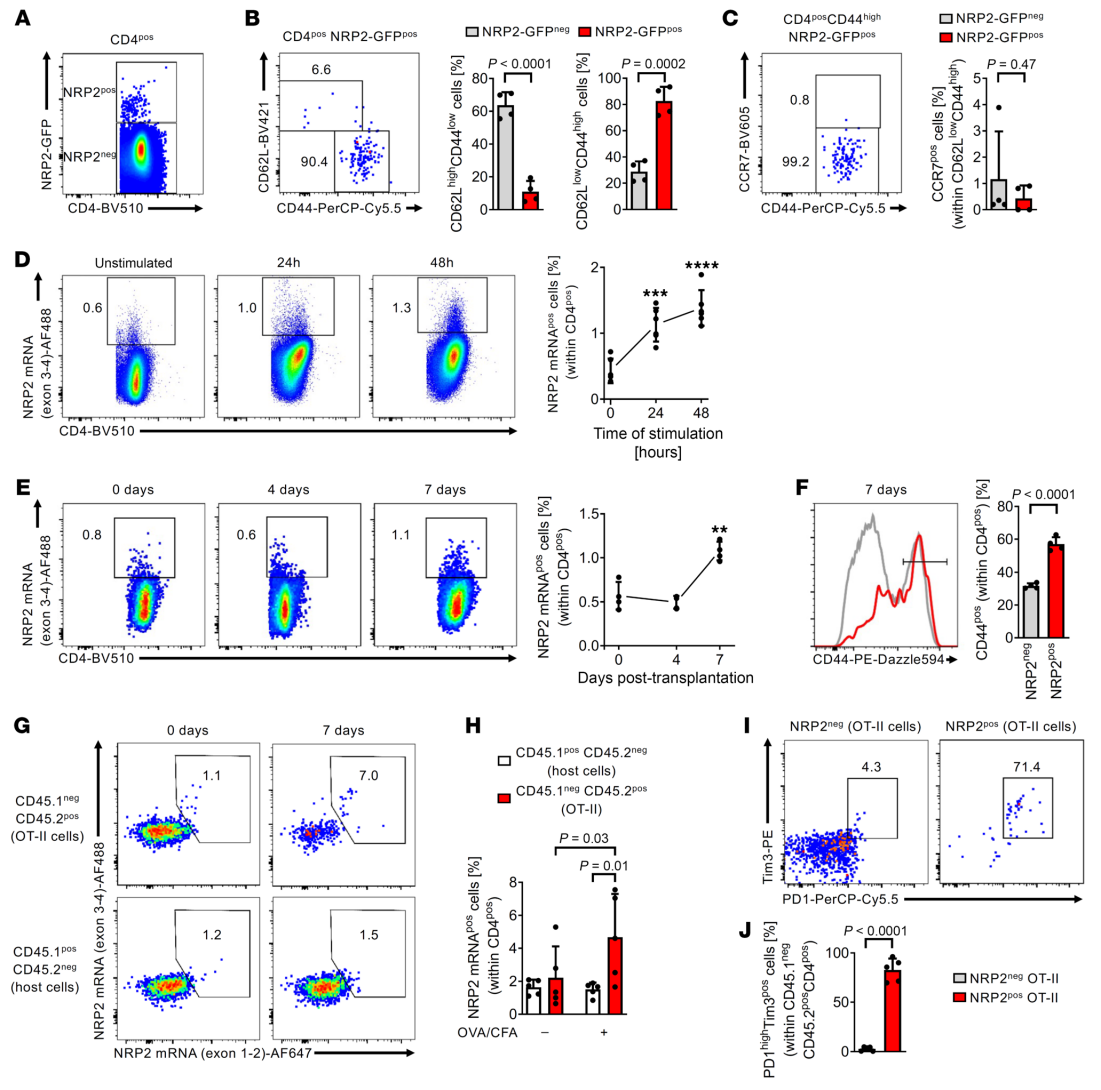
Patterns of NRP2 expression on murine CD4^+^ T cells after activation. (**A**) Representative dot plot of NRP2-GFP expression within CD4^+^ splenocytes of NRP2^lox/lox^ mice (*n* = 4 independent experiments; Supplemental Figure 5, A–D). (**B** and **C**) Representative dot plots (left) and summary of *n* = 4 independent experiments comparing phenotype of NRP2-GFP^pos^ with NRP2-GFP^neg^ CD4^+^ splenocytes. (**D**) NRP2 mRNA expression by PrimeFlow cytometry on isolated murine CD4^+^ T cells stimulated with 1 μg/mL anti-CD3 for up to 48 hours in vitro. Dot plots are gated on CD4^+^ cells. Graph illustrates changes in the expression of NRP2 mRNA in CD4^+^ T cells over 48 hours of in vitro stimulation (*n* = 6 independent experiments; mean ± SD; Kruskal-Wallis test with Dunn’s multiple-comparison test, ***P < 0.001; ****P < 0.0001 vs. unstimulated). (**E** and **F**) Fully MHC-mismatched Balb/c donor hearts were transplanted into C57BL/6 recipients. Splenocytes were isolated 4–7 days after transplant; frequency of NRP2 mRNA-expressing CD4^+^ T cells was evaluated by PrimeFlow cytometry. Nontransplanted C57BL/6 mice were included to illustrate NRP2 mRNA expression before transplant (day 0). (**E**) Dot plots are gated on CD4^+^ cells. Graph illustrates changes in expression of NRP2 mRNA in CD4^+^ T cells before and up to 7 days after transplant (*n* = 4 mice per time point; mean ± SD; Kruskal-Wallis test with Dunn’s multiple-comparison test, **P < 0.01 vs. day 0). (**F**) CD44 expression on NRP2^neg^ and NRP2^pos^ CD4^+^ T cells isolated 7 days after transplant. Graph summarizes *n* = 4 experiments (paired *t* test). (**G**–**J**) 2.5 × 10^6^ CD4^+^ T cells from CD45.2^pos^ OT-II mice were adoptively transferred into congenic CD45.1^+^ hosts by tail vein injection. Host mice were immunized s.c. with ovalbumin (50 μg) in complete Freund’s adjuvant (CFA), and the phenotype of antigen-specific OT-II and host CD4^+^ T cells were assessed after 7 days by flow cytometry. (**G**) Representative dot plots of CD45.2^pos^ OT-II (top) and CD45.1^pos^ host (bottom) CD4^+^ T cells are shown. (**H**) Mean NRP2 positivity within CD4^+^ T cells ± SD of *n* = 5/condition (2-way ANOVA with Fisher’s least significant difference test). (**I**) Representative dot plots illustrate PD1 and Tim3 expression of NRP2^pos^ (right) and NRP2^neg^ (left) OT-II CD4^+^ T cells. (**J**)Graph summarizes mean ± SD of PD1^+^Tim3^+^ cells of *n* = 5/condition (paired *t* test).

Similar to human cells (Figure 1), we found that NRP2 was expressed on a small but distinct subset of murine CD4^+^ T effector cells, and that it was inducible after mitogen-activation in vitro and allopriming in vivo (Figure 4, A–F, and Supplemental Figures 5 and 6). We also adoptively transferred ovalbumin-specific T cell receptor transgenic CD4^+^ T cells (OT-II, CD45.2) into CD45.1 hosts and profiled NRP2 expression on CD45.1 and CD45.2 cells after immunization with ovalbumin. As illustrated in Figure 4, G and H, we found a 3-fold induction in NRP2 mRNA expression (using PrimeFlow cytometry) on antigen-specific OT-II versus host CD4^+^ T cells and, consistent with our findings in Figure 2, murine NRP2^hi^ CD4^+^ T cells coexpressed PD-1 and TIM-3 (Figure 4, I and J). Also, as expected, antigen-specific NRP-2^hi^ CD4^+^ T cells were CD44^hi^CD62L^lo^, consistent with an effector/memory phenotype (data not shown).

**Figure 5:**
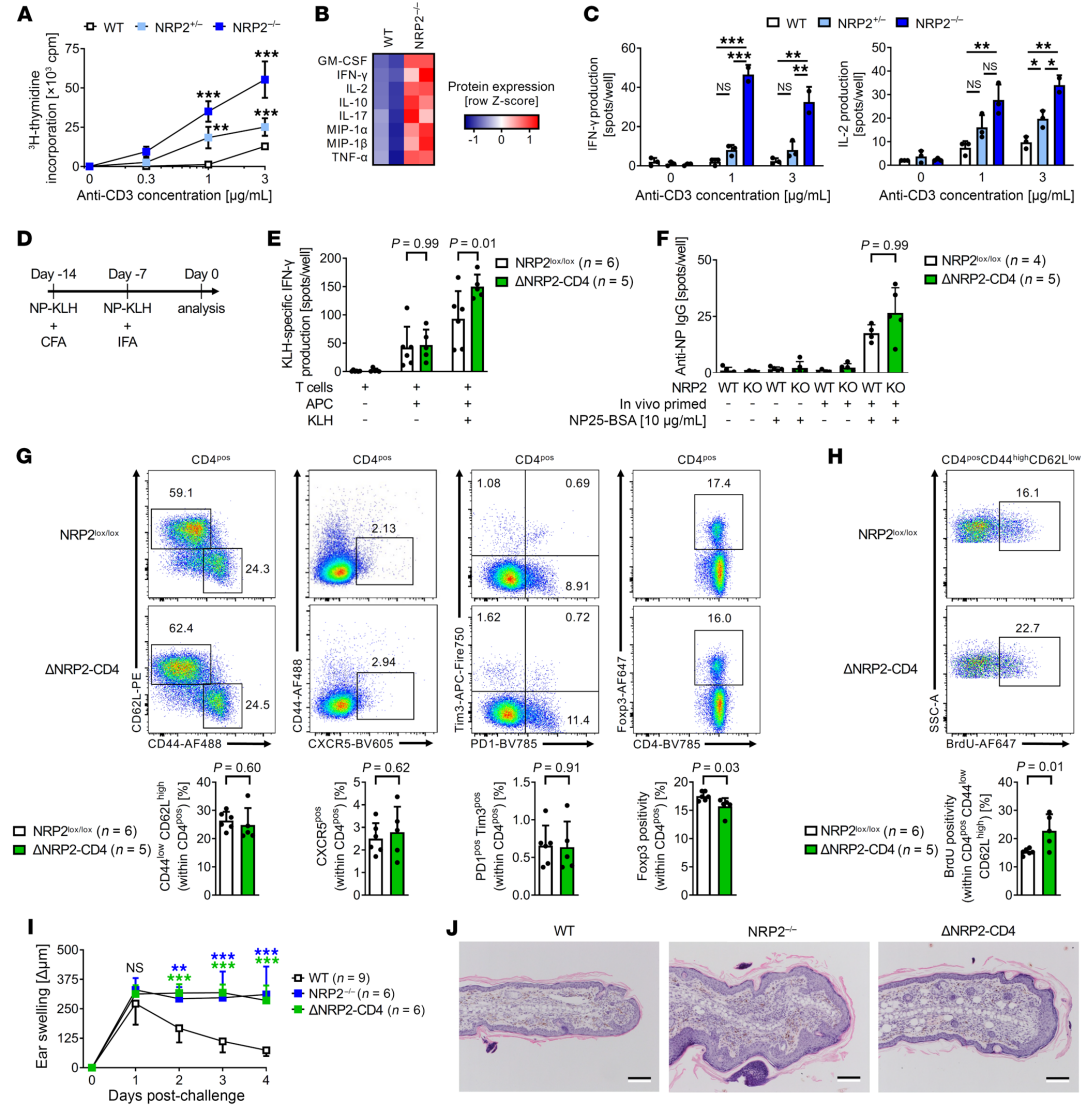
KO of NRP2 within CD4^+^ T cells increases proinflammatory responses in vitro and in vivo. (**A**–**C**) CD4^+^ T cells isolated from WT, heterozygous NRP2-KO (NRP2^+/–^) and homozygous NRP2-KO (NRP2^–/–^) mice were stimulated with increasing concentrations of plate-bound anti-CD3. (**A**) Proliferation as evaluated by ^3^H-thymidine incorporation after 72 hours (mean cpm ± SD from triplicate conditions; 1-way ANOVA, **P < 0.01, ***P < 0.001 versus WT; representative of 3 independent experiments). (**B**) Cytokine concentrations in coculture supernatants from the experiments in **A** (1 μg/mL anti-CD3) measured by multiplex-analyte profiling. Heatmap represents mean cytokine concentrations of duplicate conditions (2 independent experiments). (**C**) IFN-γ and IL-2 production as assessed by ELISPOT (mean spots ± SD of triplicate condition; 1-way ANOVA, NS not significant, *P < 0.05, **P < 0.01, ***P < 0.001 versus WT; representative of 3 independent experiments). (**D**–**H**) WT and ΔNRP2-CD4-KO mice were immunized s.c. with NP-KLH (50 μg) in CFA, boosted after 7 days with NP-KLH (50 μg) in incomplete Freund’s adjuvant (IFA), and T cell and B cell responses were analyzed after an additional 7 days. (**E**) IFN-γ production (by ELISPOT) after restimulation of primed CD4^+^ T cells to KLH. Graphs represent mean spots per well ± SD from NRP2^lox/lox^ (*n* = 6) and ΔNRP2-CD4 (*n* = 5) mice (Kruskal-Wallis test). (**F**) NP-specific IgG production in B cells by ELISPOT. Graphs represent mean spots per well ± SD from NRP2^lox/lox^ (*n* = 4) and ΔNRP2-CD4 (*n* = 5) mice (Kruskal-Wallis test). (**G**) Phenotype of splenic CD4^+^ T cell subsets. Representative dot plots (top panels) and bar graphs depicting differences between NRP2^lox/lox^ (*n* = 6) and ΔNRP2-CD4 (*n* = 5) mice (bottom panels; mean ± SD; unpaired *t* test). (**H**) Proliferation (BrdU incorporation) of CD4^+^CD44^hi^CD62L^lo^ T effector/memory cells. Representative dot plots (top panels) and graphs depicting differences between NRP2^lox/lox^ (*n* = 6) and ΔNRP2-CD4 (*n* = 5) mice (bottom panels; mean ± SD; unpaired *t* test). (**I** and **J**) WT, NRP2^–/–^, and ΔNRP2-CD4-KO mice were sensitized to oxazolone and challenged by application to the right ear in a standard DTH model; vehicle application to the left ear served as control. (**I**) Differences in thickness between right (challenge) and left (control) ears were measured daily (Δμm; 1-way ANOVA, **P < 0.01, ***P < 0.001 vs. WT). (**J**) H&E staining of challenged ears harvested on day 4 after challenge (representative of *n* = 3/condition).

To next assess the function of NRP2, we generated global NRP2-KO (NRP2^–/–^), conditional CD4-specific NRP2-KO (ΔNRP2-CD4) and Foxp3-specific NRP2-KO (ΔNRP2-Foxp3) mice. All transgenic mice were viable for more than 6 months, remained healthy, and did not develop clinical signs of autoimmunity. Furthermore, phenotyping of T cells from the thymus, lymph nodes, and spleen demonstrated normal T cell development and subset survival (Supplemental Figure 7). However, CD4^+^ T cells isolated from global NRP2^–/–^-KO and ΔNRP2-CD4-KO mice were hyperproliferative and produced increased IL-2, IFN-γ, and IL-17 after mitogenic activation in vitro (Figure 5, A–C). Furthermore, culture of CD4^+^ T cells from NRP2^–/–^ or ΔNRP2-CD4 mice in T cell differentiation media resulted in enhanced Th1 and Th2 responses and a trend toward increased Th17 responses versus WT cells (Supplemental Figure 8 and data not shown). These findings suggest a role for NRP2 in the regulation of effector CD4^+^ T cell activation.

We also evaluated antigen-specific responses in WT and ΔNRP2-CD4-KO mice after vaccination with keyhole limpet hemocyanin (NP-KLH, Figure 5, D–H). Seven days after a booster, KLH-specific CD4^+^ T cell IFN-γ responses were found to be significantly increased in ΔNRP2-CD4-KO mice versus WT mice (Figure 5E; *P* = 0.01). Anti-NP IgG B cell activity also trended higher in KO mice (Figure 5F), suggesting that NRP2 functions as a coinhibitory molecule in vivo. There was no difference in the numbers of naive CD44^lo^CD62L^hi^, CXCR5^+^ follicular (Tfh), PD-1^+^Tim-3^+^ Texh cells, and Foxp3^+^ Treg subsets after vaccination (Figure 5G). However, there was a significantly increased proliferation rate within CD44^hi^CD62L^lo^ T effector/memory cells in NRP2-KO mice compared with WT mice (Figure 5H). In vivo, antigen-specific delayed-type hypersensitivity responses also demonstrated enhanced inflammation in both global NRP2^–/–^-KO and ΔNRP2-CD4-KO mice (*P* < 0.001 vs. WT mice, Figure 5, I and J). Ear swelling peaked 24 hours after rechallenge in WT control mice and subsided over a 3-day period (Figure 5I). In contrast, the delayed-type hypersensitivity response/ear swelling failed to resolve by day 4 in KO mice, and edema and mononuclear infiltration persisted compared with WT controls (Figure 5J). Although it is possible that NRP2 may function on additional immune cell types, for example, CD8^+^ T cells in our studies, these collective findings demonstrated that it functions to regulate antigen-specific CD4^+^ T cell activation and expansion in vitro and the associated enhanced inflammatory response in vivo.

### Effector T cell responses in NRP2-KO mice after transplantation.

**Figure 6:**
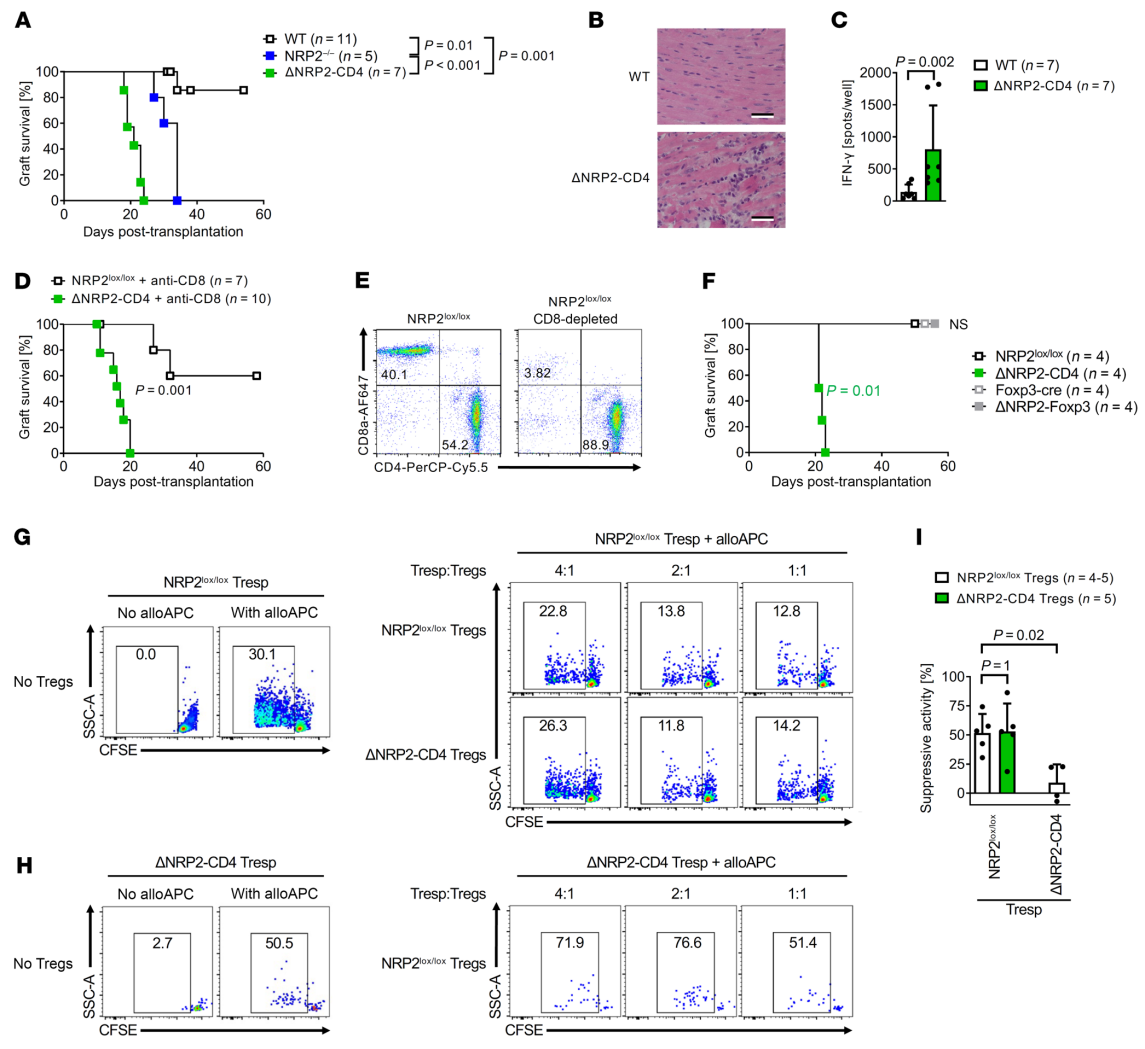
NRP2 expression on CD4^+^ T effector cells is required for long-term allograft survival. (**A**–**F**) Single MHC class II mismatched B6.C-H-2^bm12^ donor hearts were transplanted into NRP2 transgenic mice on the C57BL/6 background and graft function was monitored by palpation of the heartbeat. (**A**) Kaplan-Meier graft survival curves after transplantation of B6.C-H-2^bm12^ heart allografts into WT, NRP2^–/–^, or ΔNRP2-CD4-KO recipients. (**B**) Histology as evaluated by H&E staining of allografts harvested on day 14 after transplantation. (**C**) CD4^+^ T cell priming in WT and ΔNRP2-CD4-KO mice as evaluated by IFN-γ ELISPOT on day 14 after transplantation (mean spots/well ± SD; Mann-Whitney *U* test). (**D** and **E**) Allograft survival after transplantation of B6.C-H-2^bm12^ hearts into CD8-depleted ΔNRP2-CD4-KO or NRP2^lox/lox^ recipients. (**E**) Efficiency of CD8 depletion from splenocytes by anti-CD8 treatment peri-transplant by flow cytometry on day 2 after transplantation. (**F**) Kaplan-Meier survival curves after transplantation of B6.C-H-2^bm12^ cardiac allografts into C57BL/6 NRP2^lox/lox^, ΔNRP2-CD4, Foxp3-Cre, or ΔNRP2-Foxp3-KO recipients. (**G**–**I**) C57BL/6 NRP2^lox/lox^ and ΔNRP2-CD4-KO mice received a fully MHC-mismatched Balb/c skin transplant; Teffs and Tregs were harvested on day 14, and Treg function was assessed in an in vitro suppression assay. (**G** and **H**) A representative Treg suppression assay showing proliferation of NRP2 WT (**G**) or KO (**H**) Teff responders without Tregs (left panels) or with increasing ratios of Tregs (right panels). (**I**) Bar graph summarizing 5 independent assays comparing the percentage of suppressive activity of NRP2^lox/lox^ and ΔNRP2-CD4-KO Tregs (mean ± SD, 1-way ANOVA with Tukey’s multiple-comparison test).

We next evaluated the function of NRP2 in the regulation of alloimmunity and whether it has biological effects in CD4^+^ Teff and/or Treg responses after transplantation. Initially, we transplanted B6.C-H-2^bm12^ hearts into C57BL/6 WT or NRP2-KO mice, as this single MHC class II mismatch results in a chronic insidious response that is dependent on the relative activity of both CD4^+^ Teffs and Tregs (40, 41). As illustrated in Figure 6A, we found that graft failure was accelerated when either global NRP2^–/–^-KO mice or ΔNRP2-CD4-KO mice were used as recipients (MST = 34 days, *P* = 0.01 and MST = 21 days, *P* < 0.001, respectively) versus WT mice (MST >60 days). Also, there was a significant difference in graft survival (*P* = 0.001) when ΔNRP2-CD4-KO mice were used as recipients versus global NRP2^–/–^-KO mice, suggesting that its dominant regulatory effect in this model was related to its expression on CD4^+^ T cells (Figure 6A). Furthermore, inflammation was marked within allografts from ΔNRP2-CD4-KO recipients at early time points after transplant (Figure 6B), and the rejection response was associated with enhanced Teff priming as assessed by anti-donor IFN-γ production by recipient CD4^+^ T cells (*P* = 0.002 vs. WT recipients, Figure 6C). To confirm that rejection in ΔNRP2-CD4-KO mice is not dependent on CD8^+^ T effectors, we treated ΔNRP2-CD4-KO recipients with anti-CD8 (both before and after transplantation) and found a similar survival/rejection pattern as nondepleted mice (Figure 6, D and E, MST 17 days vs. >60 days in WT NRP2^lox/lox^ controls; *P* = 0.001). These findings suggest that the expression of NRP2 on CD4^+^ Teffs is required for long-term graft survival after transplantation.

### NRP2 expression on Tregs is redundant for the modulation of alloimmunity.

To determine whether long-term graft survival is also dependent on NRP2-expressing CD4^+^ Treg subsets in vivo, we next performed transplants using ΔNRP2-Foxp3-KO mice as recipients of B6.C-H-2^bm12^ donor hearts. As illustrated in Figure 6F, we found that allografts survived long-term in ΔNRP2-Foxp3-KO recipients (MST >60 days), similar to WT recipients. Since long-term survival in this model is dependent on the expansion and function of Tregs (40, 41), this finding indicates that NRP2 is of major significance in the regulation of CD4^+^ Teff cell activity, and that its function on Tregs is redundant.

To further confirm that Tregs are functional in the absence of NRP2, we also performed in vitro suppression assays using alloprimed Teffs and pooled populations of CD25^hi^ Tregs. Both cell types were isolated (day 14) from C57BL/6 WT or KO recipients of fully MHC-mismatched Balb/c skin transplants, and proliferation of alloreactive CD4^+^ Teffs (+/– Tregs) was evaluated in a restimulation assay after coculture with allogeneic Balb/c APCs. In this manner, suppression by either NRP2 KO or WT Tregs can be compared, as previously reported (42). As illustrated in Figure 6, G–I, and Supplemental Figure 9, we found that both NRP2-KO and WT CD25^hi^ Tregs were equally efficient in the suppression of WT effector CD4^+^ T responder cell proliferation. In contrast, we found ΔNRP2-CD4-KO T responder cell proliferation was somewhat resistant to suppression by Tregs (Figure 6, H and I). We also performed a validation experiment using FACS-sorted Foxp3-YFP^+^ Tregs instead of CD25^hi^ Tregs. Identical to the findings in Figure 6, G–I, we found that ΔNRP2-Foxp3-KO cells were functional to suppress WT T responders, and that KO T responders were resistant to Treg-mediated immunoregulation (Supplemental Figure 9, C–F). These findings confirm that NRP2-deficient Tregs are functional to suppress effector CD4^+^ T cells, and their suppressive potential is similar to WT Tregs. Our findings also suggest that NRP2-deficient Teffs are intrinsically hyperactive.

Finally, in order to inhibit Teff priming, we treated ΔNRP2-CD4-KO or WT NRP2^lox/lox^ recipients of fully MHC-mismatched Balb/c hearts with costimulatory blockade (anti-CD154 on days 0 and 2 after transplantation). We confirmed inhibition of allogeneic priming using a standard restimulation assay involving coculture with allogeneic Balb/c APCs (Supplemental Figure 10, A and B). There was an almost identical graft survival pattern in KO and WT recipients after anti-CD154 treatment (Supplemental Figure 10C), suggesting that NRP2 was functional via its biology on alloactivated Teff subsets. Similar to the B6.C-H-2^bm12^ model, graft survival after anti-CD154 treatment may also be dependent on Treg activity (43, 44). However, our observations demonstrated that Treg function was normal in the absence of NRP2, both in vivo (Figure 6F) and in vitro (Figure 6I), further indicating that the lack of difference in survival in this model was related to the inhibition of Teff priming/expansion.

### Independence of NRP2 and PD-1/PD-L1 coinhibition.

**Figure 7:**
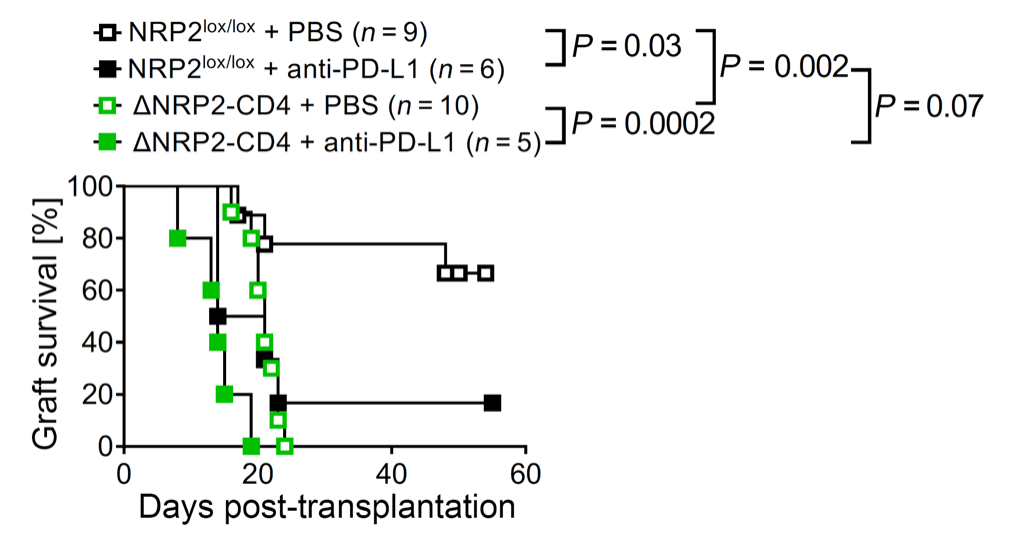
The regulation of allograft rejection by CD4^+^ T cell NRP2 is independent of PD-1/PD-L1. Kaplan-Meier graft survival curves after the transplantation of B6.C-H-2^bm12^ heart allografts into ΔNRP2-CD4 or NRP2^lox/lox^ recipients that were treated with either anti–PD-L1 or PBS (on days 0, 3, and 6 after transplantation).

As discussed above, we found that NRP2 is coexpressed with known immune checkpoint/coinhibitory molecules on phenotypically exhausted human and mouse CD4^+^ T cells. Since PD-1 is implicated in T cell dysfunction after transplantation (5), we also sought to determine whether the regulatory effects of CD4^+^ T cell NRP2 are dependent on its association with PD-1. B6.C-H-2^bm12^ mice were used as donors and C57BL/6 WT or ΔNRP2-CD4-KO mice were used as recipients, either untreated or treated with anti–PD-L1. We found that anti–PD-L1 treatment of WT recipients resulted in accelerated rejection and early graft failure (MST = 17.5 days; *P* = 0.02) versus PBS-treated WT NRP2^lox/lox^ controls (MST >60 days; Figure 7). Anti–PD-L1 also reduced graft survival in ΔNRP2-CD4-KO recipients (MST = 14 days) versus in PBS-treated KO mice (MST = 21 days; *P* = 0.0002, Figure 7), and it resulted in a trend for accelerated rejection in ΔNRP2-CD4-KO mice versus WT recipients (*P* = 0.07, Figure 7). We interpret these observations to indicate that the coinhibitory function of NRP2 is elicited through distinct signaling responses and/or regulatory ligands, independent of its association with PD-1.

## Discussion

Immunoregulatory mechanisms that promote long-term graft survival after solid organ transplantation are not fully understood. In this report, we found that NRP2 is expressed on a subset of human and mouse immune cell lineages, including T cells, B cells, and APCs. We also found that it functions as a cell surface coinhibitory receptor on CD4^+^ T cells to regulate cell-mediated and alloimmune responses. Furthermore, it is induced after vaccination on antigen-activated effector/memory CD4^+^ T cells, and its level of expression peaks on cells that are phenotypically and functionally exhausted. KO of NRP2 is associated with enhanced activation and proliferation in vitro, and enhanced inflammation in vivo in models of antigen-induced immunity and allograft rejection. Since NRP2 binds multiple ligands, these findings indicate that the relative expression of NRP2 on CD4^+^ T cell subsets has broad implications for the regulation of inflammatory disease.

Our previous studies demonstrated that the NRP2 receptor elicits regulatory signaling responses in multiple cell types (including T cells) via the inhibition of PI3K/Akt/mTOR activity (45). Here, we found that KO of NRP2 results in hyperactive CD4^+^ T cell responses in vitro, including enhanced IL-2 and IFN-γ production after mitogen activation and augmented T helper subset differentiation. These findings are consistent with the well-established function of PI3K/mTOR signaling in effector CD4^+^ cell responses (46–51). However, although activation of the mTOR signaling pathway inhibits Treg-dependent biology, we failed to observe any effect of NRP2 deficiency on the suppressive activity of Tregs in vitro or in vivo. Since NRP1 may also be expressed on Treg subsets (21, 33, 34, 36, 52), it is possible that NRP2-induced signaling is redundant inasmuch as both receptors elicit similar regulatory responses via common mechanisms, for example, via the cross-linking of Plexin A family molecules (18, 23, 24, 45, 53–56). Thus, the loss of NRP2-dependent inhibition of Akt/mTOR signaling (45) may be compensated for by ligand-dependent cross-linking of Plexin A by NRP1 (53). Redundancy also explains why deletion of the NRP1 gene alone on Foxp3^+^ Tregs does not result in autoimmune disease (34). Notably, in our studies, deletion of NRP2 did not result in any clinical signs of autoimmunity, and phenotyping of KO mice demonstrated normal T cell development (Supplemental Figure 7). Thus, the lack of autoimmunity in KO mice is likely related to the redundancy of NRP2 on Tregs. Consistent with this interpretation, we note that this phenotype is similar to other coinhibitory molecule KOs that do not develop autoimmunity but mount hyperactive antigen-specific immune responses (57). Although beyond the scope of the current studies, these observations suggest that the combined effects of NRP1 and NRP2 are necessary for Treg function which is critical for long-term transplant survival, the prevention of autoimmune disease, and/or tumor immunity.

We also questioned why NRP2 was expressed by a small subset of CD4^+^ T cells at baseline, but deletion resulted in a generalized expansion after antigen-induced stimulation. One possibility is that NRP2 functions to limit the expansion of Teffs by promoting clonal deletion of Texh, an established mechanism that results in prolongation of graft survival (43). But deletion alone in the absence of immunoregulation is not necessarily sufficient to promote long-term graft survival (43). The expansion of Teffs in vivo in NRP2-deficient recipients may also be the result of a lack of Treg-dependent immunoregulation (40) (58). However, as discussed above, we found that NRP2-KO Tregs function as efficiently as WT Tregs in the suppression of primed alloresponsive Teffs. In contrast, NRP2-deficient Teffs appear to be intrinsically hyperactive and are resistant to Treg-mediated suppression. Since NRP2 is induced on expanded populations of CD44^hi^ effector/memory CD4^+^ T cells, we interpret our findings to indicate that its main function is to augment clonal deletion of CD4^+^ Texh cells and/or to promote dysfunction. Consistent with this possibility, we also found that NRP2^pos^ cells are functionally characterized by reduced rates of proliferation and reduced cytokine production as compared with NRP2^neg^ T cells. Furthermore, they express a transcriptional program that includes multiple coinhibitory receptors that augment exhaustion/dysfunction (4, 59, 60). Interestingly, it was reported that IFN regulatory factor 4 (IRF4) modulates CD4^+^ T cell differentiation into Texh (5, 61–64), and transcriptional data reveal that IRF4-KO CD4^+^ T cells express NRP2 (5). Indeed, the NRP2 promoter has 5 putative PU.1/IRF4 binding sites, and pilot analyses indicated that IRF4–NRP2 interactions are indeed inhibitory (data not shown). Thus, it is possible that when IRF4 is expressed (for example, after early activation), it serves as a common mechanism to inhibit the expression of NRP2 and prevent the initiation of exhaustion. We thus suggest that the initial activation of CD4^+^ T cells results in IRF4-induced transcriptional activity that represses NRP2 expression; once IRF4 activity decreases, repression of NRP2 is removed, allowing for ligand-dependent augmentation of T cell dysfunction and clonal deletion.

NRP2 ligands are broadly expressed within different tissues and may be functionally associated with the regulation of cell-mediated immune inflammation. For example, TGF-β is well established to be immunomodulatory (65), whereas VEGF-A and VEGF-C are generally associated with proinflammation (66, 67). Semaphorin family members are expressed within tumors, where they are generally associated with immune evasion (39, 68–70). Pathologically, VEGF-A may bind NRP2 as an accessory receptor, where it competitively occupies the semaphorin binding site and prevents semaphorin-dependent inhibitory signaling. In this manner, high levels of VEGF-A within a tissue, including allografts undergoing chronic rejection (67, 71, 72), may inhibit immunomodulation induced by semaphorin-NRP2 interactions. In contrast, high levels of semaphorins (for example, within tumors) and/or soluble NRP2 that is present in the circulation (16, 73, 74) may competitively inhibit VEGF-VEGFR–induced signaling and proinflammation (66). In our studies, we found a survival difference when either global NRP2 KOs or our CD4^+^ T cell KOs were used as recipients. We interpret this finding to indicate that NRP2 is also functional on other immune cell types (Figure 1, A and B, and Supplemental Figure 5, B–D) and/or that soluble NRP2 functions as a scavenger receptor to inhibit rejection, for example, by inhibiting proinflammatory VEGF-A (54, 66, 73).

NRP2 ligands have potential to be expressed within allografts (72, 75), we performed an exploratory analysis of ligand expression using a public dataset of human renal transplant biopsies (Supplemental Figure 11) (76, 77). This analysis revealed that SEMA3F was among the highest expressed genes in stable/normal biopsies from patients on a tolerance induction protocol (Supplemental Figure 11B). These intriguing observations suggest that augmentation of the local expression of NRP2 ligands within allografts (including SEMA3F) may serve to sustain immunoregulation after transplantation.

In summary, NRP2 is expressed on a subset of antigen-activated effector/memory CD4^+^ T cells and notably on CD4^+^ Texh cell subsets, where it functions to regulate alloimmunity and rejection after transplantation. It is also expressed on Tregs, but its function is dominantly related to its biology on CD4^+^ Teffs. We conclude that NRP2 signaling within CD4^+^ T cell subsets has implications for effector alloimmunity and thus long-term survival after transplantation.

## Methods

### Sex as a biological variable.

Both males and females were used in all human and mouse studies, but donor-recipient sex mismatch was generally avoided in murine transplant studies.

### Isolation and cell culture of human PBMCs and CD4^+^ T cells.

Human PBMCs were isolated from healthy adult volunteer donors by high-density centrifugation of blood (Corning). CD4^+^ T cells were enriched and negatively isolated from human PBMCs using magnetic isolation beads (Stemcell Technologies) and purity of greater than 98% was determined by flow cytometry. Cells were cultured in RPMI 1640 (Lonza) supplemented with 10% FBS (MilliporeSigma), 2 mM L-glutamine, 1 mM sodium pyruvate, 0.75 g/L sodium bicarbonate, 100 U/mL penicillin/streptomycin, 0.1 mM nonessential amino acids (all Lonza), and 50 μM 2-mercaptoethanol (MilliporeSigma) and activated using PHA (1–10 μg/mL; Remel, Thermo Fisher Scientific).

### Isolation and cell culture of murine CD4^+^ T cells.

Murine CD4^+^ T cells were negatively isolated from splenocytes as previously described (78). Cells were cultured in RPMI 1640 (Lonza) supplemented with 10% FBS (MilliporeSigma), 2 mM L-glutamine, 1 mM sodium pyruvate, 0.75 g/L sodium bicarbonate, 100 U/mL penicillin/streptomycin, 0.1 mM nonessential amino acids (all Lonza), and 50 μM 2-mercaptoethanol (MilliporeSigma). Murine T cells were stimulated with plate-bound anti-CD3 (clone 145-2C11; BioXcell), and proliferation was assessed by ^3^H-thymidine incorporation (1 μCi/well during last 16 hours of culture; scintillation counter: Wallac 1450 MicroBeta TriLux, version 4.6, PerkinElmer). Cytokine production was evaluated by either ELISPOT using the Ready-Set-Go Enzyme-linked immunospot assay kit (Thermo Fisher Scientific) according to the manufacturer’s instructions, or by multiple analyte profiling using the mouse cytokine/chemokine 25 plex magnetic bead panel (MilliporeSigma) on a Luminex LX200 platform equipped with xPonent software (version 3.1.871.0). T helper cell polarization was performed as previously described (78). Briefly, naive CD4^+^ T cells were activated with plate-bound anti-CD3 and soluble anti-CD28 in polarizing conditions for 48 hours and rested in the absence of anti-CD3 for an additional 48 hours. Th differentiation was subsequently evaluated by stimulation with 10 μg/mL concanavalin A (Cayman Chemical Company) in IFN-γ (Th1), IL-4 (Th2), or IL-17A (Th17) ELISPOT assays (all Thermo Fisher Scientific).

### Flow cytometry.

Cells were washed twice in PBS supplemented with 0.5% FBS and 6 μM EDTA (Boston BioProducts), preincubated with FcR blocker (BioLegend) for 20 minutes, stained with fluorochrome-conjugated antibodies (Supplemental Table 1) for 60 minutes at 4°C, washed, and analyzed within 24 hours. Specificity of anti-human NRP2 antibody staining was established using 2 different clones and in knockdown experiments (Supplemental Figure 12). Briefly, U87MG cells (ATCC) were cultured in EMEM supplemented with 10% FBS (MilliporeSigma) and 100 U/mL penicillin/streptomycin (Lonza) as described (45). NRP2 knockdown was performed with siRNA or scramble siRNA using lipofectamine in Opti-MEM (all Thermo Fisher Scientific) for 24 hours; after washing, cells were cultured for an additional 3 days before analysis according to our previously reported protocol (45). The specificity of commercial anti-murine NRP2 antibodies for use in cytometry were evaluated using murine NRP2-KO cells (and confirmed by mRNA and Western blot analysis) and referenced to analysis using an NRP2-GFP-tag in transgenic mice. In general, we had concern that antibodies bind nonspecifically to NRP2-KO cells. Thus, for all flow-based studies, we used a single-cell mRNA fluorescence in situ hybridization technique (PrimeFlow, Thermo Fisher Scientific) to evaluate NRP2 expression on immune cell subsets. PrimeFlow assays were performed using 2 independent probes targeting either exon 1–2 or exon 3–4 of the NRP2 transcript. Target specificity was confirmed using NRP2-KO cells (Supplemental Figure 6C). As a technical control, we used a probe targeting CD4 mRNA, and probe amplification in each sample was confirmed by comparing signals of CD4 mRNA versus protein (not shown). We also used the GFP-tag in NRP2^lox/lox^ mice to identify NRP2 expression, but analysis of GFP was technically difficult due to the low signal/autofluorescence ratio of the GFP-tag. FoxP3 staining was performed using a FoxP3/transcription factor staining buffer set (Thermo Fisher Scientific). Intracellular cytokine staining was performed using an intracellular staining permeabilization buffer (BioLegend) on cells that were activated with 50 ng/mL phorbol 12-myristate 13-acetate and 1 μg/mL ionomycin (both MilliporeSigma) in the presence of 5 μg/mL brefeldin A (BioLegend) for 6 hours. BrdU staining was performed according to the manufacturer’s instructions using the phase-flow kit (BioLegend). Stained cells were analyzed either on a FACS Calibur (2 lasers), Celesta (3 lasers), or LSR II (5 lasers) cytometer (all BD Biosciences) and data were evaluated using FlowJo software (version 10.7, Tree Star). Cell sorting was performed on a FACS Aria II or Aria Fusion (both BD Biosciences) to a purity of greater than 95%. Gating strategies and purity controls are depicted in each experiment.

### Immunocytology.

For immunocytological analysis, CD4^+^ T cells were immobilized on ImmunoSelect adhesion slides (MoBiTec) for 20 minutes in PBS at 37°C. Adherent cells were subsequently fixed in 4% paraformaldehyde in PBS (Boston BioProducts) for 15 minutes at room temperature. After a wash in PBS, cells were permeabilized in cold methanol for 5 minutes at –20°C, washed, and incubated with blocking buffer (5% BSA in PBS) for 30 minutes at room temperature and subsequently with primary antibodies (Supplemental Table 1) overnight at 4°C. After washing in PBS, cells were incubated with a species-specific fluorochrome-conjugated secondary antibody (Thermo Fisher Scientific) for 1 hour at room temperature, washed, and mounted with ProLong Gold antifade containing 4′,6-diamidino-2-phenylindole (Thermo Fisher Scientific). Staining was imaged on a confocal laser-scanning microscope (TCS SP5 X, Leica) equipped with LAS AF software (version 1.6.3, Leica). Specificity of the NRP2 signal was evaluated using isotype control stainings.

### Western blot analysis.

Western blot analysis was performed using standard techniques and as previously described (78). Briefly, cells were lysed in RIPA buffer supplemented with EDTA and run on a 12% SDS–polyacrylamide gel (Bio-Rad). Proteins were transferred to a PVDF membrane (MilliporeSigma) and blocked with 10% BSA in TBS supplemented with 0.1% Tween 20 (TBST, Boston BioProducts). Primary antibodies (see Supplemental Table 1) were diluted in blocking buffer prior to incubation overnight at 4°C. After 3 washing steps in TBST, membranes were subsequently incubated with species-specific peroxidase-conjugated secondary antibodies (Jackson ImmunoResearch) for 1 hour at room temperature. After washing, the protein of interest was detected by chemiluminescence (Thermo Fisher Scientific) using a ChemiDoc MP imaging system (Bio-Rad). Membranes were stripped and reprobed to control for equal protein loading.

### PCR.

PCR was performed using standard techniques. Briefly, DNA was extracted using the hot-alkaline DNA extraction method. Regions of interest were amplified using specific primer sets (Supplemental Table 2) and a master mix containing Taq DNA polymerase, deoxynucleotide triphosphates (dNTPs), and reaction buffer according to the manufacturer’s instructions (Promega). PCR products were run on an agarose gel containing SYBR Green and visualized using a GelDoc XR+ imaging system (Bio-Rad).

### Transcriptomic analyses.

For bulk RNA-Seq, cells were sorted by flow cytometry staining, pelleted, lysed in TRIzol (Thermo Fisher Scientific), and stored at –80°C for group analysis. Library preparations using poly-A–enriched RNA and sequencing was performed by Genewiz. Paired-end 150 bp reads were aligned to a reference human genome database (GRCh38) using the 2 pass STAR aligner (79), and gene expression levels were quantified using the GENCODE gene models (release M14; ref. 80) using the HTSeq count method (81). Normalization and differential gene expression were determined using the DESeq2 method version 1.28.1 (82) in R version 4.0.0. Functional enrichment, signaling pathway activities, and protein association network analyses were performed using the gene set enrichment (83) and STRING (84) bioinformatics resources. One sample was identified as an outlier using principal component analysis and subsequently removed from the analysis.

Single-cell RNA-Seq was combined with detection of NRP2 protein expression in a CITE-Seq assay. Briefly, cells were preincubated with 10 μg/mL streptavidin and FcR blocker (both BioLegend) for 20 minutes, washed twice, stained with fluorochrome-conjugated antibodies for cell sorting, and PE-conjugated anti-NRP2 primary antibody (clone MM03, Sino Biologics) and biotin-conjugated anti-NRP2 primary antibody (clone 2v2, a gift from ATyr Pharma) (Supplemental Table 1). Cells were washed and incubated with DNA-conjugated anti-PE and anti-biotin secondary antibodies (BioLegend) (Supplemental Table 1). After washing, cells were FACS-sorted, washed again, and resuspended in PBS supplemented with 1% BSA. Next, 3′-end mRNA and CITE-Seq library preparations were immediately performed on a 10x Chromium platform (10x Genomics) and sequenced on a NextSeq 500 (Illumina). FASTQ files were demultiplexed and aligned to the human genome (GRCh38) using the Cell Ranger pipeline (version 3.1.0, 10x Genomics) and imported into Seurat version 3.2.0 (85) in R version 4.0.0. Cells with unusually high unique molecular identifiers (doublets), mitochondrial gene percentages (dying cells), and CD8^+^ T cells were excluded from further analysis. Predefined immune gene (86) expression was log-normalized, dimensional reduced, and clustered based on k-nearest neighbors. Markers for each cluster were ranked by their log-fold change, and cluster annotation was manually chosen based on gene expression profiles. Trajectory-based pseudotime analysis was performed using the Monocle3 method, version 0.2.2 (87).

### Mouse strains.

The following mice were purchased from the Jackson Laboratory: 6–10-week-old C57BL/6 WT (CD45.2^+^) and congenic *Ptprc^a^Pepc^b^* (CD45.1^+^, both H-2b), Balb/c (H-2d), and B6.C-H-2^bm12^. Ovalbumin-specific T cell receptor transgenic (OT-II) mice were gifted by Hans Oettgen (Boston Children’s Hospital, Boston, MA) and originally purchased from the Jackson Laboratory. SCID-beige mice were purchased from Taconic. Transgenic NRP2-KO and NRP2-floxed mice were purchased from the Jackson Laboratory and backcrossed to greater than 98% congenicity using C57BL/6 mice (384-SNP panel, Charles River Laboratories). NRP2-floxed mice were crossed with C57BL/6 CD4-Cre (Taconic) and Foxp3-Cre (the Jackson Laboratory) mice to generate conditional KO strains (ΔNRP2-CD4 and ΔNRP2-Foxp3, respectively). For each experiment, NRP2^lox/lox^ mice and 100% congenic C57BL/6 mice (the Jackson Laboratory) were included as controls.

### HuSCID-beige mice.

HuSCID-beige mice were generated by the transfer of 5 × 10^7^ human PBMCs by tail vein injection. At a time course (generally up to 21 days) after humanization, mice were euthanized and the spleens were harvested, dissociated into a single-cell solution, blocked with human and mouse FcR blocker, and stained with fluorochrome conjugated antibodies as described above (see Supplemental Table 1). The phenotype of human CD4^+^ T cells within the spleen was assessed by gating on live human CD3^+^CD4^+^ cells and gating out mouse CD45^+^ cells using flow cytometry. Alternatively, human CD4^+^ cells were FACS-sorted from splenocytes using the same gating strategy (human CD3^+^CD4^+^ cells/mouse CD45^neg^) for transcriptomic analysis.

### Heart transplantation.

Heterotopic intraabdominal cardiac transplantation was performed as previously described (88) and graft survival was monitored by palpation of the heartbeat. In some recipients, CD8^+^ T cells were depleted by i.p. injection of anti-CD8 (200 μg of clone 53-6.7; BioXcell) on day –5, –2 and +2 peritransplant and every fourth day thereafter. Efficacy of depletion was evaluated by flow cytometry. As indicated, some recipients were treated with anti-CD154 (200 μg/i.p.; clone MR-1, BioXcell) on days 0 and 2 after transplantation or anti–PD-L1 (200 μg/i.p.; clone 10F.9G2, BioXcell) on days 0, 3, and 6 after transplantation.

### Treg suppression assays.

Allogeneic priming of T cells was performed by tail skin transplantation as we described previously (78) using Balb/c donors and C57BL/6 WT or ΔNRP2-CD4-KO mice as recipients. Spleens were harvested on day 14 after transplantation and CD4^+^CD25^hi^ Tregs and CD4^+^CD25^neg^ T responders were isolated as described above, and subsequently stained with CFSE (Thermo Fisher Scientific) prior to coculture with irradiated (1200 rad) Balb/c splenocytes stained with CellTrace Far Red (Thermo Fisher Scientific). Tregs from WT or ΔNRP2-CD4-KO recipients were added to cocultures in increasing ratios with T responders (1:16 to 1:1), and suppression of T responder proliferation was evaluated after 5 days by flow cytometry. For statistical purposes, suppressive activity was calculated as ([percentage of CFSE^dim^ cells in the absence of Tregs] – [percentage of CFSE^dim^ cells at 1:1 T responders:Tregs])/[percentage of CFSE^dim^ cells in the absence of Tregs].

### Priming/antigen-induced responsiveness in vivo.

Allogeneic priming of CD4^+^ T cells after cardiac transplantation was assessed by coculture of recipient CD4^+^ T cells (isolated from recipient splenocytes) in a mixed lymphocyte reaction with irradiated (1200 rad) donor splenocytes. Recipient CD4^+^ activity was either evaluated using CFSE dilution after 5 days of coculture or by IFN-γ ELISPOT using anti–IFN-γ coated 96-well PVDF ELISPOT plates (Immobilon-P; MilliporeSigma). After 24 hours coculture, ELISPOT plates were washed twice with 0.1% Tween 20 in PBS and subsequently incubated with a biotinylated anti–IFN-γ antibody (BioLegend, catalog 505704) (Supplemental Table 1). After 1 hour at 4°C, ELISPOT plates were washed twice with 0.1% Tween 20 in PBS and subsequently incubated with HRP-conjugated avidin for 45 minutes. After 2 washing steps with 0.1% Tween 20 in PBS and then PBS, HRP-activity was developed using 3-amino-9-ethylcarbazole (AEC, BD Biosciences). Stained plates were scanned and analyzed on an ImmunoSpot S6 Ultra ELISPOT reader (version 5.0, CTL).

In some experiments, ovalbumin-specific T cell receptor transgenic OT-II CD4^+^ T cells (2.5 × 10^6^ cells/mouse) were adoptively transferred by tail vein injection into congenic C57BL/6 Ptprc^a^Pepc^b^ (CD45.1^pos^CD45.2^neg^) host mice. After 24 hours, host mice were immunized s.c. in the right flank with 50 μg ovalbumin in CFA (InvivoGen). Subsequently, NRP2 expression and the phenotype of antigen-specific (identified using anti-CD45.2) versus host (identified using anti-CD45.1) CD4^+^ T cells were evaluated in splenocytes at a time course up to 7 days after vaccination.

Antigen-induced responsiveness was assessed after vaccination, where mice were immunized with 50 μg NP-KLH (NP/KLH ratio, 24:1; LGC Biosearch Technologies) emulsified in CFA s.c. in the right flank. After 7 days, the mice received a booster of NP-KLH (50 μg) emulsified in incomplete Freund’s adjuvant. After an additional 7 days, the mice were euthanized, and CD4^+^ T cells and CD19^+^ B cells were purified from the spleen by negative selection using magnetic bead isolation kits (Stemcell Technologies). In vivo priming to KLH was assessed by in vitro stimulation of CD4^+^ T cells with KLH for 24 hours and IFN-γ production was assessed by ELISPOT, as described above. B cells were stimulated in NP-conjugated BSA (1 μg/mL; ratio, 25:1; LGC Biosearch Technologies) in coated 96-well PVDF plates for 24 hours, and responses were assessed by ELISPOT after incubation with 4 μg/mL HRP-conjugated anti-mouse IgG for 1 hour at 4°C (Supplemental Table 1). As above, plates were washed both before and after secondary antibody incubation and prior to development using AEC (BD Biosciences). In some experiments, 1 mg BrdU (BioLegend) was i.p. injected every 12 hours for the last 72 hours and proliferation as well as T cell subset phenotype were analyzed using flow cytometry.

### Delayed-type hypersensitivity.

Delayed-type hypersensitivity responses were performed using an established model (89). Briefly, mice were sensitized using 2% 4-ethoxymethylene-2-phenyl-2-oxazolin-5-one (oxazolone; dissolved in 20% olive oil in acetone; MilliporeSigma) applied to the shaved abdomen (50 μL) and each paw (5 μL). Five days after sensitization, delayed-type hypersensitivity responses were evaluated in the right ear after reapplication of a 1% oxazolone solution (10 μL); the left ear was treated with vehicle alone as a control. The thicknesses of the ears was measured daily with a caliper (dial thickness gauge; Swiss Precision Instruments) and is expressed as the difference between oxazolone-challenged versus vehicle control (Δμm).

### Histology.

Tissue was harvested and fixed in 10% formaldehyde in PBS overnight at 4°C, paraffin-embedded, sectioned, and stained with H&E (all Thermo Fisher Scientific). Staining was imaged on a Nikon Eclipse 80i using a Retiga-2000R CCD camera (QImaging) equipped with NIS Elements software (version 3.22.15; Nikon).

### Statistics.

Statistical analyses were performed using a 2-tailed Student’s *t* test or 1-way ANOVA as indicated, with previous testing of equality of variances. A Mann-Whitney *U* test or Kruskal-Wallis test was used if variances were significantly different. Graft survival was analyzed using a log-rank test. *P* values of less than 0.05 were considered significant. Heatmaps were generated using the heatmap.2 function in the gplots package (version 3.0.4) using R version 4.0.0.

### Study approval.

Volunteer blood donors provided consent in accordance with IRB approval at Boston Children’s Hospital. All animal studies were approved by the IACUC at Boston Children’s Hospital and complied with the *Guide for the Care and Use of Laboratory Animals* (National Academies Press, 2011).

### Data availability.

Raw datasets generated in this study are available in the NCBI’s Gene Expression Omnibus (GEO) repository under the accession number GSE231735. Transcriptomic data from renal graft biopsies were reanalyzed from publicly available data (GSE106675). The graphical abstract was created with BioRender.com.

## Author contributions

JW and DMB conceptualized the study and designed the experiments. JW, NK, SB, JL, MM, BA, KM, KL, HZ, MK, and HN conducted experiments, and JW, NK, MM, BA, and KM acquired data. JW, NK, SWK, and DMB analyzed data. DRB provided reagents and mice and supported data analysis. JW and DMB wrote and edited the manuscript.

## Supplementary Material

Supplemental data

Unedited blot and gel images

Supporting data values

## Version 1 07/01/2025

Electronic publication
