# Supplementary material for: Neuropilin-2 functions as a coinhibitory receptor to regulate antigen-induced inflammation and allograft rejection
Source: J Clin Invest. 2025 Jul 1;135(13):e172218. doi: 10.1172/JCI172218 (PMC12208552; doi:10.1172/JCI172218)
Supplement: Supplemental data [file jci-135-172218-s093.pdf]

Supplemental Material

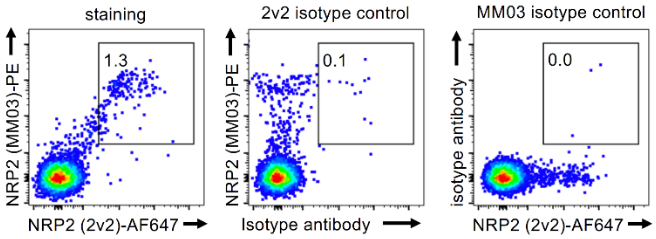

Supplemental Figure 1: Specificity of NRP2 flow cytometric staining was evaluated using freshly isolated PBMC and isotype control antibodies. Dot plots show $CD4^+$ T cells (gated).

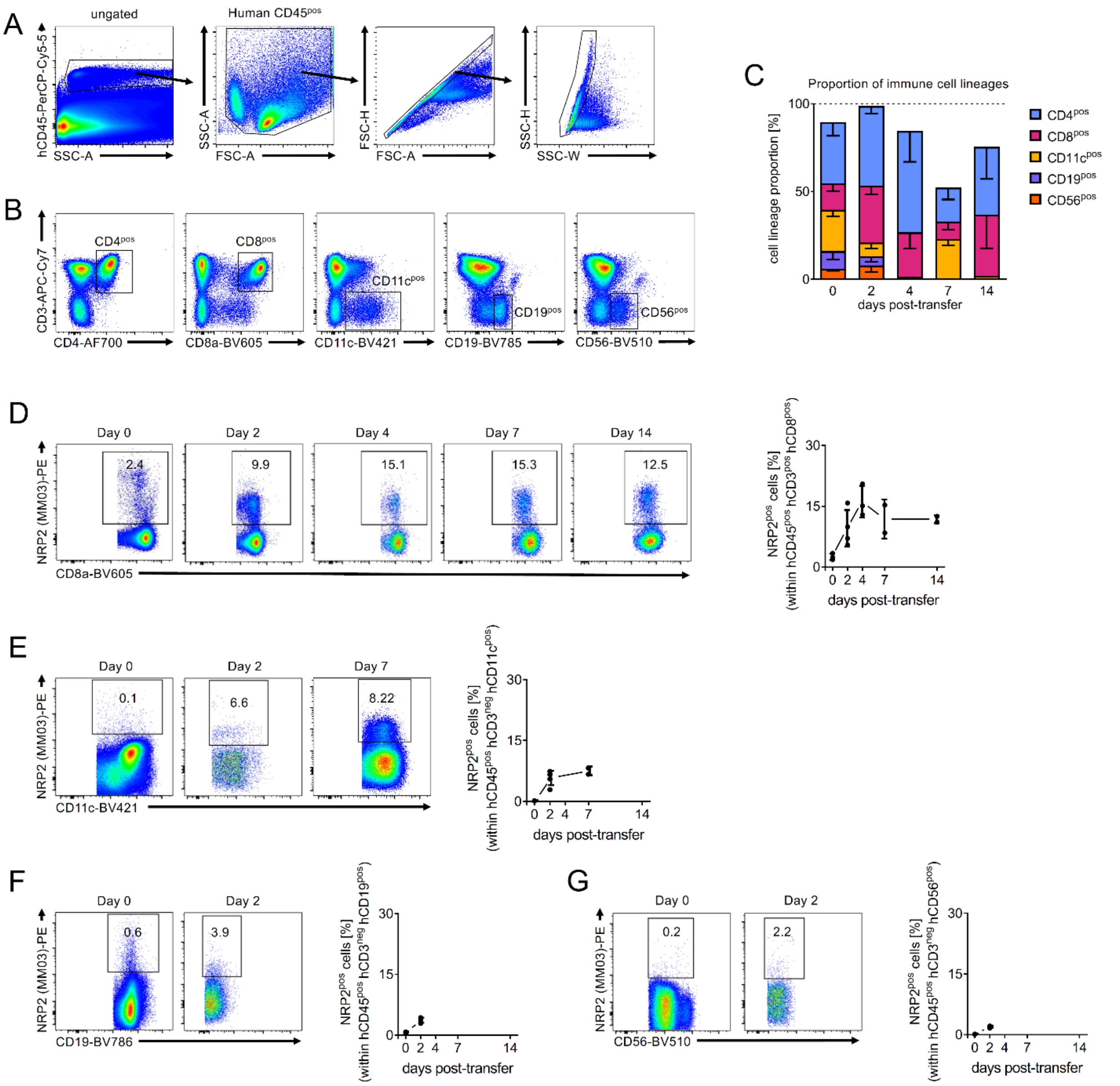

Supplemental Figure 2: Analysis of NRP2 expression on human immune cells within huSCID mice. **(A)** Gating stragtegy for the identification of human cells from huSCID mice (also depicted in Figure 1F-G). **(B)** Identification of human immune cell lineages isolated from splenocytes of huSCID mice. Dotplots are gated on human CD45^{pos} cells. **(C)** Bar graph represents the percent of each lineage and lineage perisitence within human CD45^{pos} cells at different time-points post-transfer in SCID-beige mice. NRP2 expression on **(D)** human CD8^{+} T cells, **(E)** human CD11c^{+} myeloid cells, **(F)** human CD19^{+} B cells, and **(G)** human CD56^{+} NK cells for up to 14 days dependent on survivial within the huSCID. Representative dot plots are gated on human CD45^{+} and each lineage marker^{+}. Line graphs illustrate a summary of NRP2 expression in n=2-4 independent experiments per time-point.

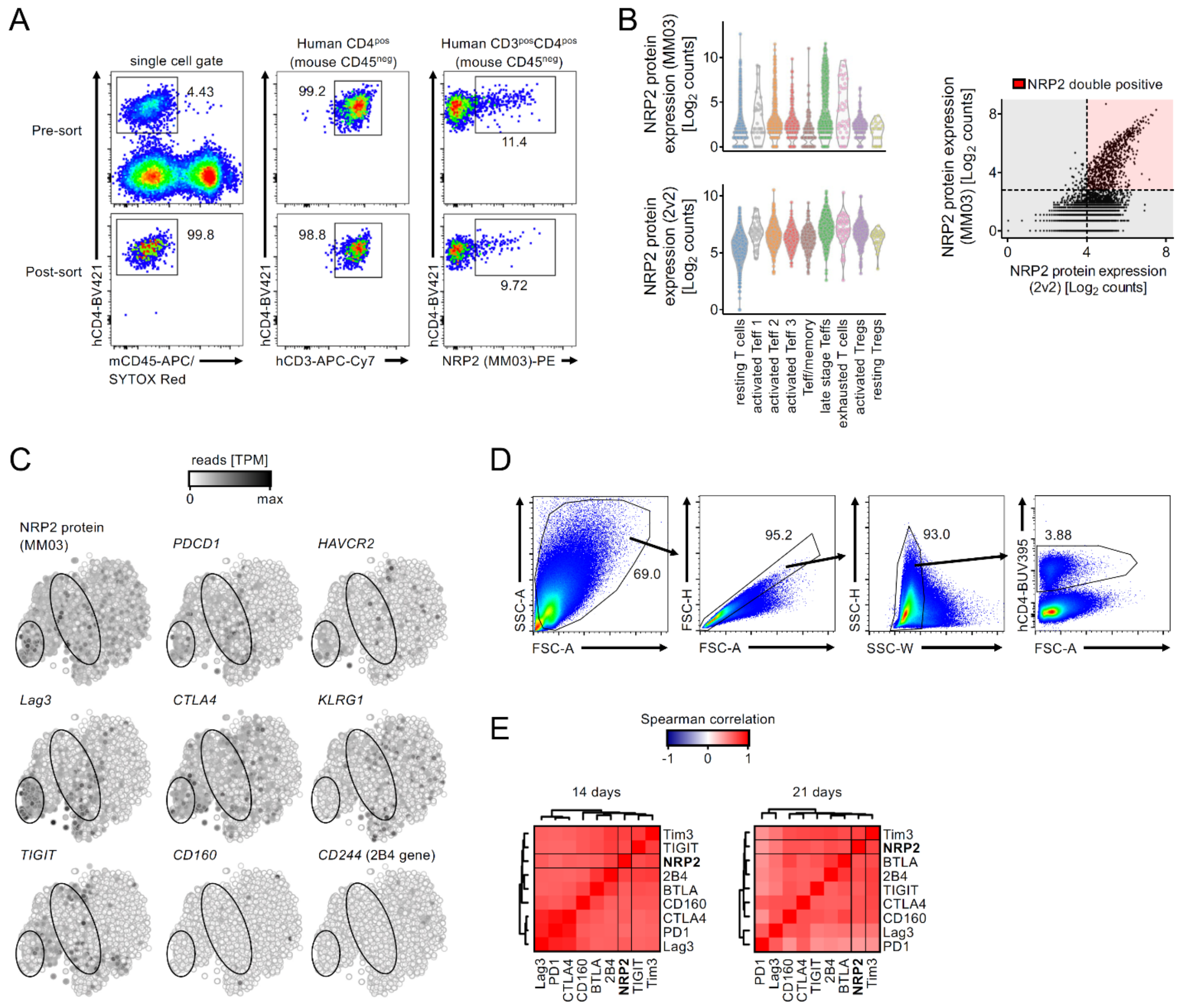

Supplemental Figure 3: Gating strategies and raw data used in Figure 2. (A) Sorting strategy and purity control for sorting of human CD3$^+$CD4$^+$ T cells from the huSCID model on day 7 post transfer (mCD45: mouse CD45; hCD3: human CD3; hCD4: human CD4). Single cell gating, and hCD4 cells in which mCD45 is gated out (CD45^{neg}) are shown. **(B)** Left: Violin blots of sequenced NRP2 protein expression on the cells identified in Panel A using CITE-seq (top: clone MM03; bottom: clone 2v2). Right: Scatter plot of scRNA-seq NRP2 antibody counts. Color highlighted areas define NRP2^{pos} and NRP2^{neg} cells for each antibody. Positivity using both antibodies is highlighted in red. **(C)** tSNE plots (grey-scale highlighted) of co-inhibitory receptor mRNA expression and NRP2 protein expression (using the MM03 clone). NRP2 enriched regions are highlighted by circles (see Figure 2A-B). **(D)** Gating strategy for flow cytometric analysis of human CD4$^+$ T cells used in Figure 2E-J. **(E)** Flow cytometric analysis was performed on CD4$^+$ T cells harvested from the huSCID model on day 7 (Figure 2E), day 14 and day 21 post humanization. Heatmaps were generated to illustrate the mean spearman's rank correlation coefficient between the expression of NRP2 and co-inhibitory receptors (n=4 independent experiments per time-point).

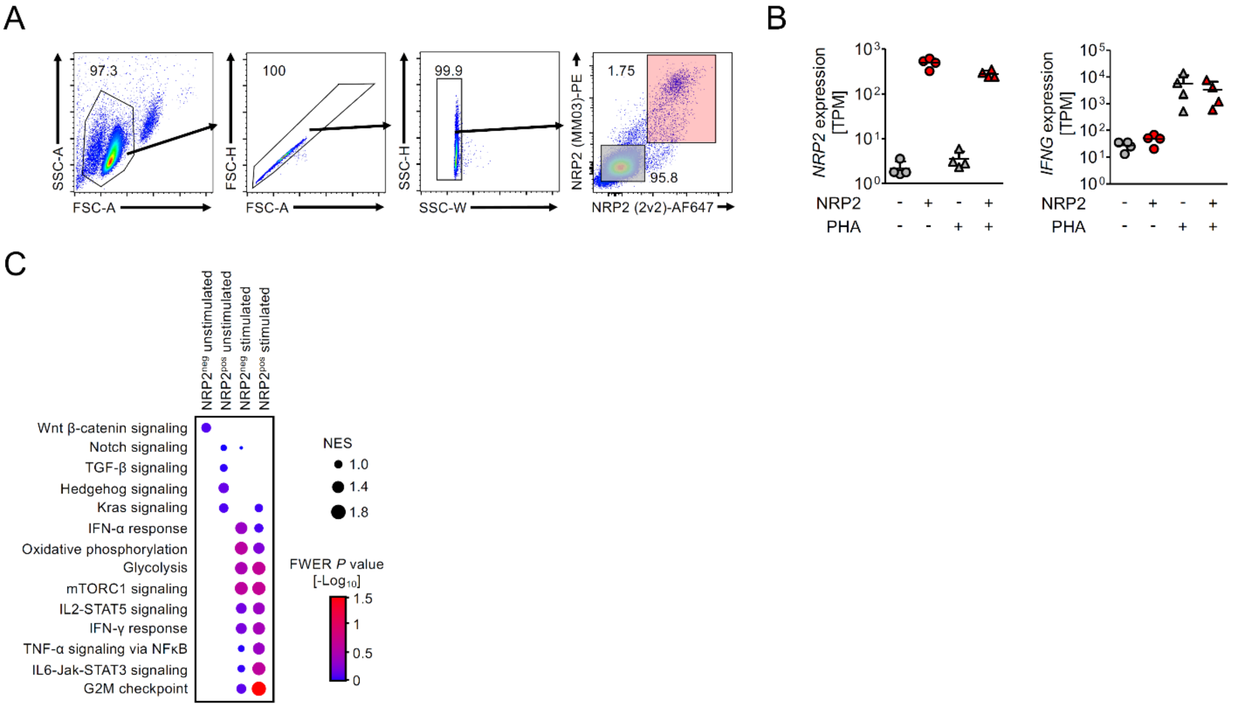

Supplemental Figure 4: Gating strategy and extended analysis of bulk RNA-seq data. (A) Sorting strategy for the purification of NRP2^{pos} and NRP2^{neg} cells. **(B)** *NRP2* and *IFNG* mRNA expression in different conditions as quality controls for sorting and mitogen activation respectively. **(C)** Dot plot representing normalized enrichment scores (NES) and familywise error rate adjusted (FWER) *P* values for the gene set enrichment analysis. Signaling pathway activities of cells from four conditions NRP2^{pos} and NRP2^{neg} cells either unstimulated or PHA-stimulated (3 µg/ml for 16 hours) was estimated using the Hallmark gene set database.

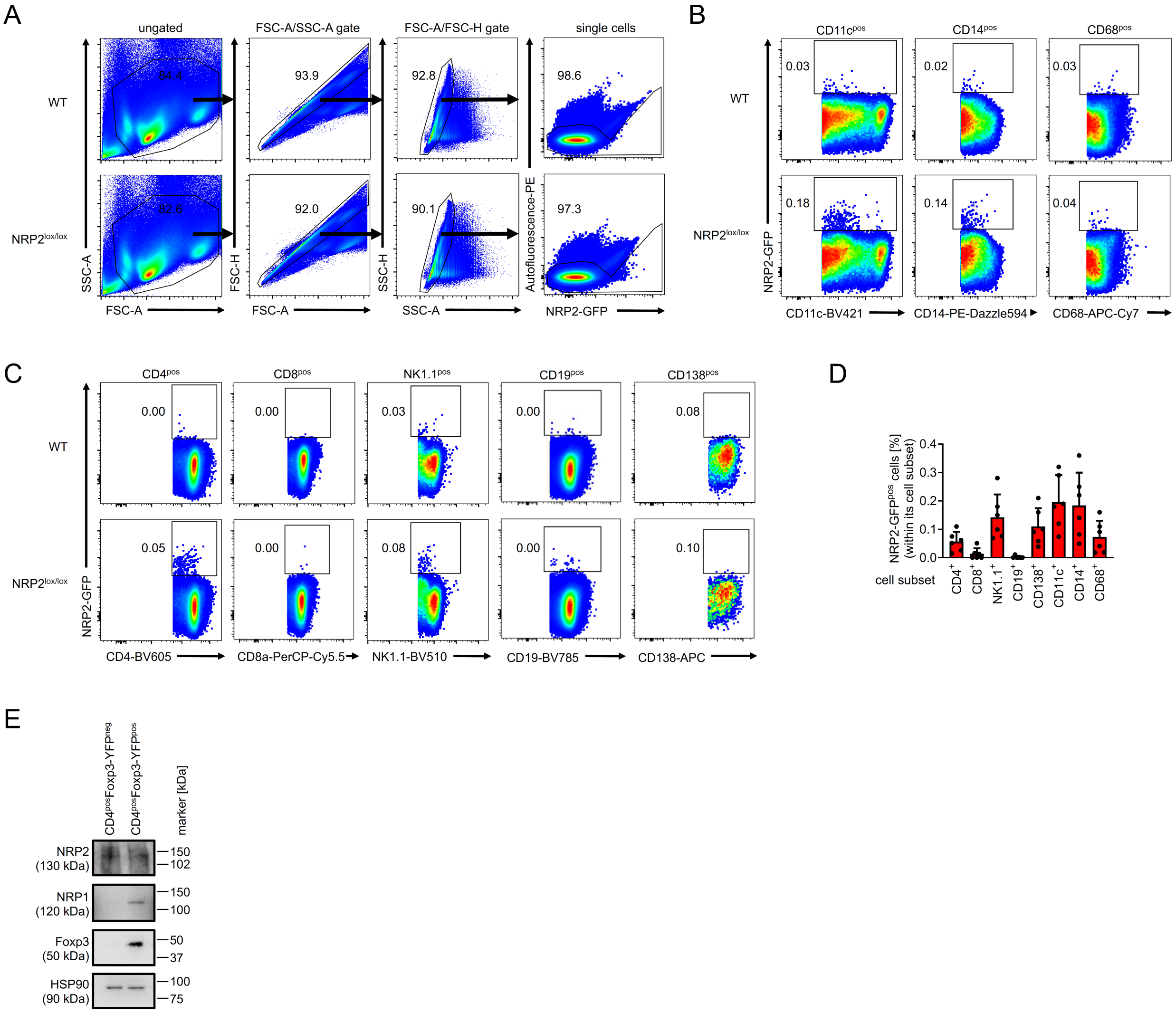

Supplemental Figure 5: NRP2 expression within murine splenocytes. (A) Gating strategy to identify NRP2-GFP expression in splenocytes. WT C57BL/6 mice were used to distinguish autofluoresence from specific NRP2-GFP signals in NRP2$^{lox/lox}$ mice. **(B-C)** Representative dot plots and **(D)** a summary of n=6 independent experiments of NRP2-GFP expression within splenocyte subsets. **(E)** Western blot analysis of FACS-sorted CD4^{+}Foxp3-YFP^{pos} (Treg) and CD4^{+}Foxp3-YFP^{neg} (Teff) cells isolated from splenocytes of Foxp3-cre mice. Representative of n=4 independent experiments.

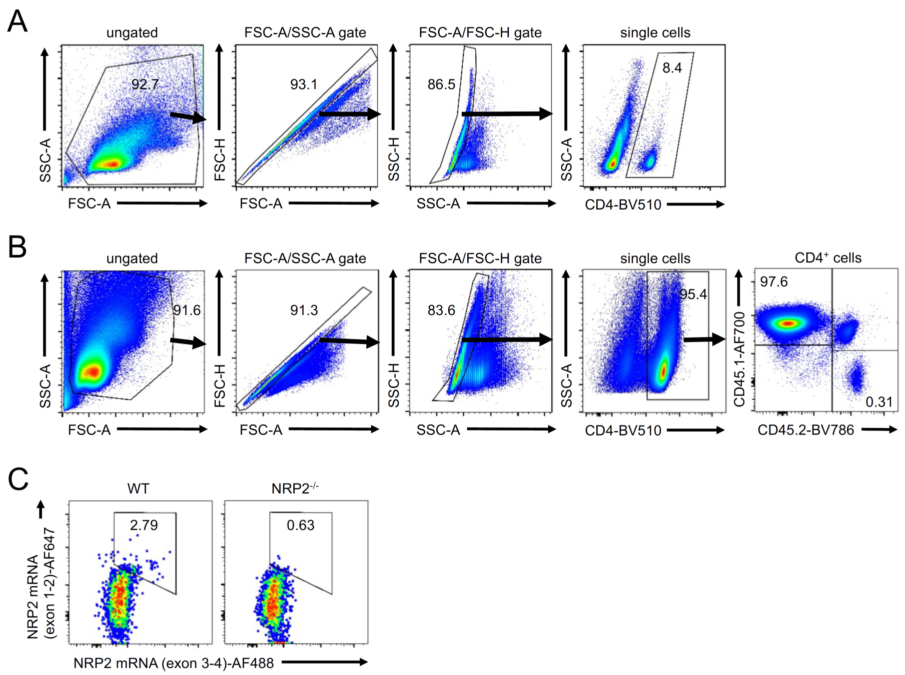

Supplemental Figure 6: Gating strategy and controls for PrimeFlow assays. (A) Gating strategy to identify CD4$^+$ T cells depicted in Figure 4D-F. **(B)** Gating strategy to identify CD45.1^{pos} CD45.2^{neg} host and CD45.1^{pos} CD45.2^{pos} OT-II CD4$^+$ T cells (depicted in Figure 4G-J). **(C)** Splenocytes from C57BL/6 WT and NRP2$^{-/-}$ were stained in the same experiment to identify the gate for NRP2 mRNA positivity. Dot plots show the positive (left: WT splenocytes) and negative (right: NRP2 KO splenocytes) technical controls for the fluorescence in situ hybridization.

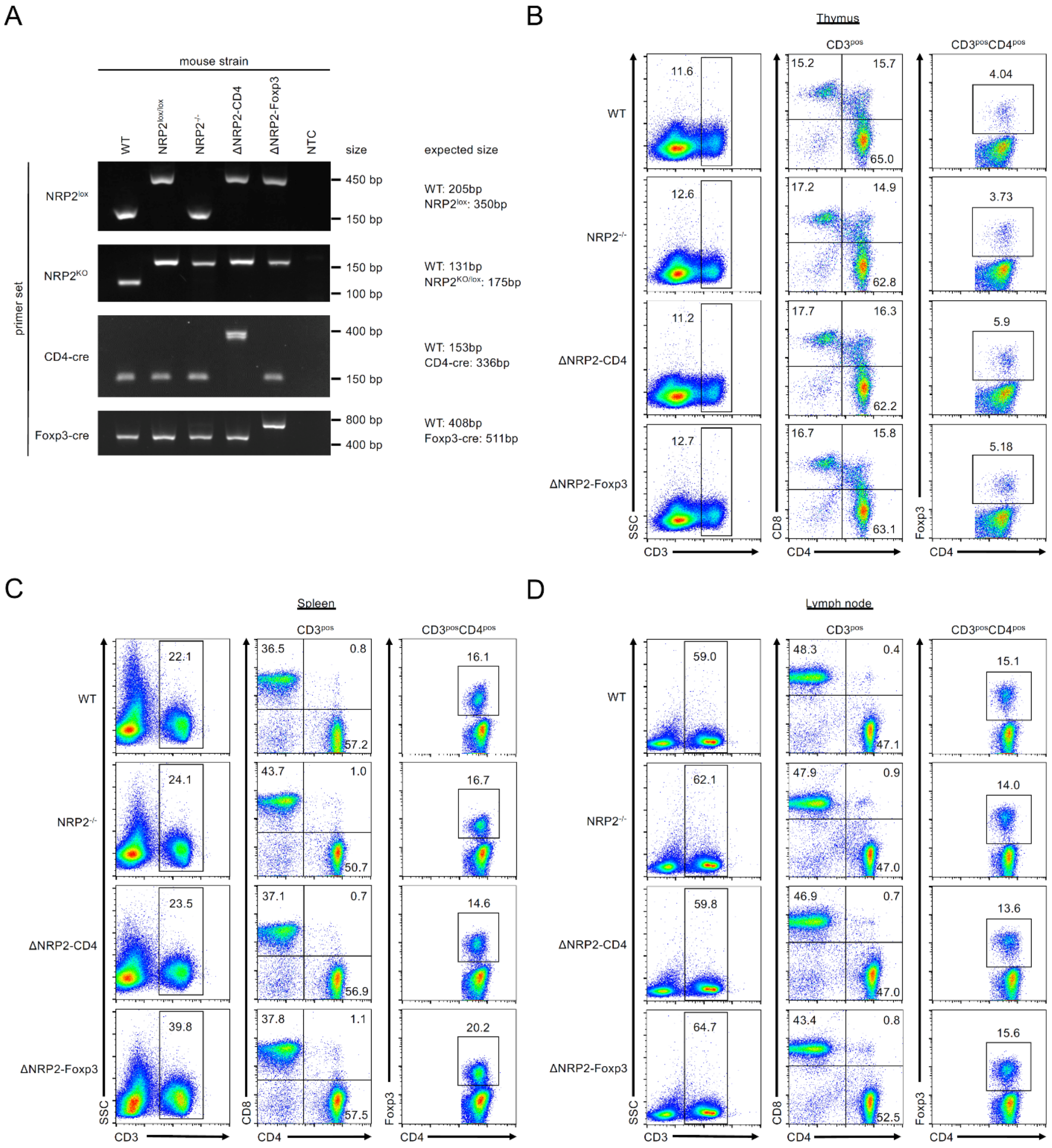

Supplemental Figure 7: Phenotyping of transgenic mice. (A) Genotyping of C57BL/6 WT, global NRP2$^{-/-}$ knockout, conditional ΔNRP2-CD4 knockout and ΔNRP2-Foxp3 knockout mice (DNA from tail biopsies). **(B-D)** Immunophenotyping of WT and transgenic mice by flow cytometry. Dot plots are representative of n=3 mice initially evaluated. NRP2$^{lox/lox}$ and Foxp3-cre mice were similar to WT mice (not shown). Frequencies of CD3, CD4, CD8 and Foxp3 positive cells in the **(B)** thymus **(C)** spleen and **(D)** inguinal lymph nodes.

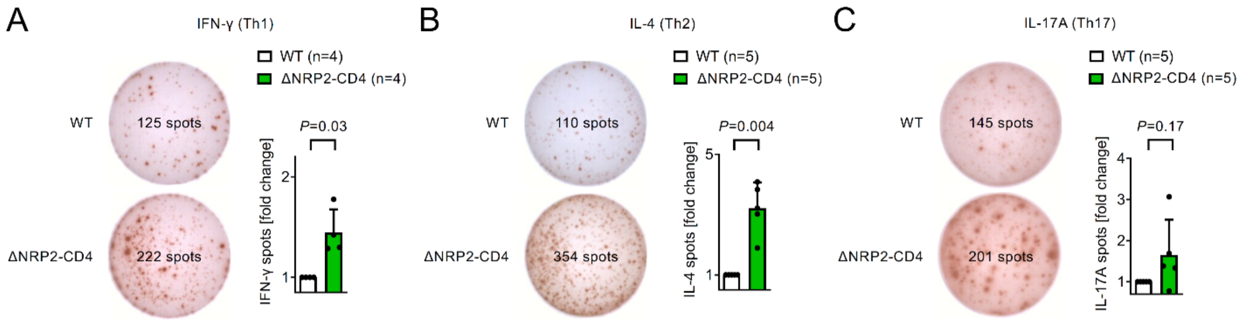

Supplemental Figure 8: Knockout of NRP2 in murine CD4$^+$ T cells increases T helper cell differentiation *in vitro*. Naive CD4$^+$ T cells from either WT or ΔNRP2-CD4 knockout mice were cultured in T helper cell (Th) polarizing conditions for 48 hours as described (78), rested for an additional 48 hours, and **(A)** Th1, **(B)** Th2 and **(C)** Th17 differentiation was evaluated using ELISPOT assays respectively. Representative images of ELISPOT wells (left) and bar graphs (from n=4-5 experiments, right) summarizing the fold change in IFN-γ, IL4 and IL-17A comparing knockout cells to WT cells (fold change of mean spots/well ± SD; One sample t-test).

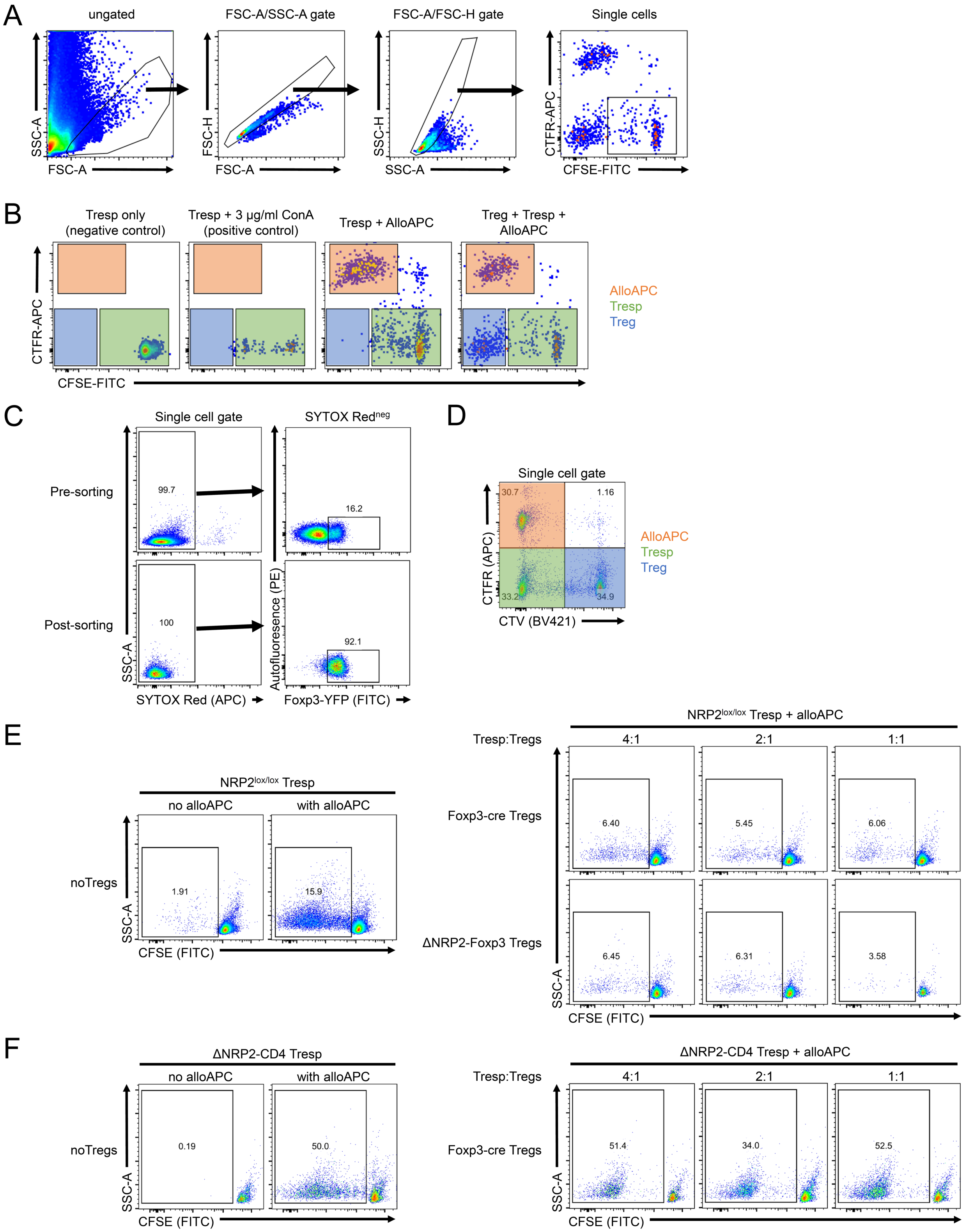

Supplemental Figure 9: Gating strategy and controls for Treg suppression assays. C57BL/6 NRP2$^{lox/lox}$ and ΔNRP2-CD4 knockout mice received a fully MHC mismatched Balb/c skin transplant; Teff and Treg were harvested on day 14 and in vitro suppression assays were performed

following coculture with irradiated Balb/c APCs as in Figure 6G-I. **(A)** Gating strategy and **(B)** controls to identify a Tresp gate (highlighted in green). The same gating strategy and controls were used for Treg suppression assays in Figure 6G-H. **(C-F)** Validation in vitro suppression experiment using purified populations of Foxp3-YFP Tregs (complimentary to data shown in Figure 6G-I). C57BL/6 NRP2$^{lox/lox}$, ΔNRP2-CD4, Foxp3-cre and ΔNRP2-Foxp3 mice received a fully MHC mismatched Balb/c skin transplant. On day 14 post-transplant, CD4^{+}Foxp3-YFP^{+} Tregs were FACS-sorted from Foxp3-cre and ΔNRP2-Foxp3 mice and stained with CellTrace Violet (CTV). CD4^{+}CD25^{neg} Tresp were harvested from NRP2$^{lox/lox}$ and ΔNRP2-CD4 recipients and stained with CFSE. Treg function was assessed in an in vitro suppression assay as depicted in Figure 6G-I using irradiated and CellTrace Far Red (CTFR)-stained Balb/c splenocytes (alloAPC; Tresp:alloAPC ratio 1:1). **(C)** Dotplots depict cell viability and Foxp3 positivity pre- and post-sorting. **(D)** Representative dotplot illustrates the gating strategy to identify CTV^{neg}CFSE^{pos}CTFR^{neg} Tresp cells for evaluating Tresp proliferation using CFSE dilution. **(E-F)** Proliferation of NRP2 WT (E) and NRP2 KO (F) Tresp without Tregs (left panels) or with increasing ratios of either NRP2 WT (right panel; top row) of KO (right panel; bottom row) Tregs. One validation experiment was performed.

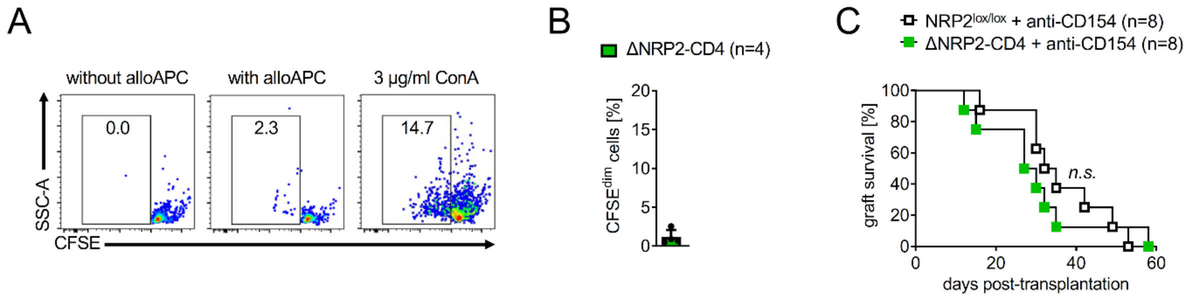

Supplemental Figure 10: Costimulatory blockade using anti-CD154 inhibits allogeneic priming of Teffs and abrogates NRP2 function. (A-B) Fully MHC mismatched Balb/c hearts were transplanted into NRP2$^{lox/lox}$ wild type or ΔNRP2-CD4 knockout recipients treated with anti-CD154 on days 0 and 2 post transplantation. Allogeneic priming on day 14 post-transplantation was evaluated by coculture of recipient CD4^{+} T cells with irradiated donor Balb/c splenocytes for 5 days; proliferation as assessed by CFSE dilution. Concavalin A (ConA, 3 µg/ml) was used as a positive control. **(A)** Representative dot plots and **(B)** bar graph of n=4 mice is depicted (frequency of alloreactive ΔNRP2-CD4 knockout CFSE^{dim} cells ± SD)**. (C)** Kaplan-Meier graft survival curves (*n.s.* not significant).

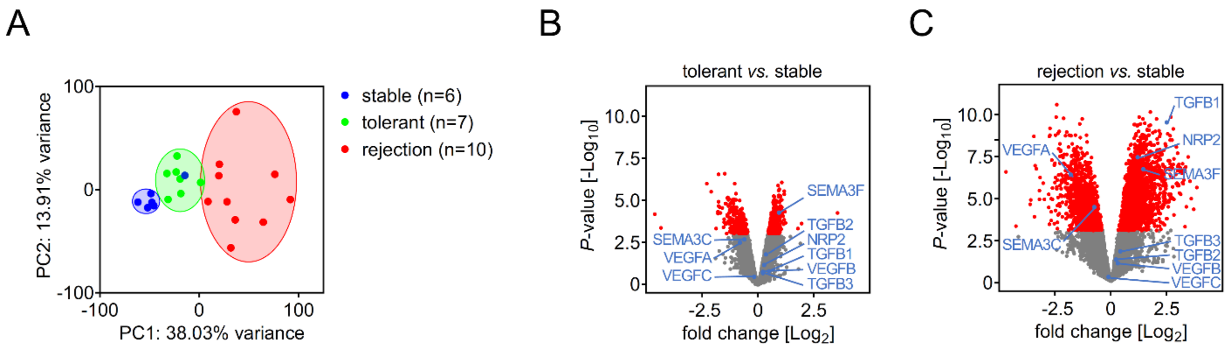

Supplemental Figure 11: Expression of NRP2 ligands in tolerant renal transplant biopsies. We analyzed a published database of renal transplant biopsies (GSE106675) that were taken from renal transplant recipients following tolerance induction using the Northwestern protocol (76-77). The database also included findings from surveillance biopsies from a control cohort of stable transplants recipients on conventional immunosuppression, and from patients with clinical rejection. **(A)** Principal component analysis of all samples. **(B-C)** Volcano plot analysis comparing stable patients on immunosuppression to **(B)** biopsies from tolerant patients off immunosuppression or **(C)** biospies with evidence of rejection. Statistically significant (P<0.001) regulated transcripts are highlighted in red, and NRP2 ligands are highlighted as blue dots.

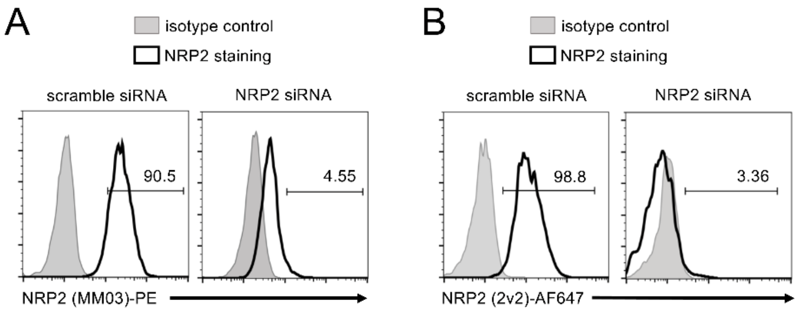

Supplemental Figure 12: NRP2 antibody specificity. The specificity of the two anti-human NRP2 antibody clones used in this study was assessed in a knockdown approach using the NRP2-expressing U87MG glioblastoma cell line as previously described (45). **(A)** Binding of the MM03 anti-NRP2 clone, and **(B)** binding of the 2v2 anti-NRP2 clone to scramble siRNA (left panels) or NRP2 siRNA (right panels) transfected U87MG cells.

Supplemental Table 1. Antibodies used in these studies.

target (fluorochrome/enzyme/DNA-oligo)	clone	company
anti-Biotin (A0436 [DNA])	1D4-C5	Biolegend
anti-BrdU (AF488, AF647)	3D4	Biolegend
anti-human 2B4 (AF700)	C1.7	Biolegend
anti-human BTLA (PE-Cy5)	MIH26	Biolegend
anti-human CD11c (BV421)	3-HCL-3	Biolegend
anti-human CD160 (PE)	51-10C9	BD Biosciences
anti-human CD19 (BV785)	HIB19	Biolegend
anti-human CD279/PD-1 (BV421)	NAT105	Biolegend
anti-human CD3 (unconjugated, APC-Cy7)	HIT3a	Biolegend
anti-human CD38 (BV605)	HB-7	Biolegend
anti-human CD366/Tim3 (APC-Cy7)	F38-2E2	Biolegend
anti-human CD4 (BUV395)	RPA-T4	BD Biosciences
anti-human CD4 (BV421, APC, AF700)	OKT4	Biolegend
anti-human CD45 (PerCP-Cy5.5)	HI30	Biolegend
anti-human CD56 (BV510)	5.1H11	Biolegend
anti-human CD69 (BV421)	FN50	Biolegend
anti-human CD8a (BV605)	HIT8a	Biolegend
anti-human CTLA4 (BV605)	BNI3	Biolegend
anti-human HLA-DR (AF488)	L243	Biolegend
anti-human IFNγ (AF488)	4S.B3	Biolegend
anti-human Lag3 (BV510)	11C3C65	Biolegend
anti-human NRP2 (biotin, AF647)	2v2	aTyr Pharma
anti-human NRP2 (unconjugated, PE, APC)	MM03	Sino Biologics
anti-human TIGIT (PE-Cy7)	A15153G	Biolegend
anti-human TruStain FcX	---	Biolegend
anti-mouse CD11c (BV421)	N418	Biolegend
anti-mouse CD138/Syndecan-1 (APC)	281-2	Biolegend
anti-mouse CD14 (PE-Dazzle594)	Sa14-2	Biolegend
anti-mouse CD19 (BV785)	6D5	Biolegend
anti-mouse CD197/CCR7 (BV605)	4B12	Biolegend
anti-mouse CD279/PD1 (BV785, PerCP-Cy5.5)	29F.1A12	Biolegend
anti-mouse CD3 (PE)	17A2	Biolegend
anti-mouse CD366/Tim3 (PE, APC-Fire750)	B8.2C12	Biolegend
anti-mouse CD4 (BV510, BV605, BV785, PerCP-Cy5.5, AF700)	GK1.5	Biolegend
anti-mouse CD44 (AF488, PerCP-Cy5.5)	IM7	Biolegend
anti-mouse CD45 (APC)	30-F11	Biolegend

anti-mouse CD45.1 (BV510, AF700)	A20	Biolegend
anti-mouse CD45.2 (BV786, PE-Dazzle594)	104	Biolegend
anti-mouse CD62L (BV421, PE)	MEL-14	Biolegend
anti-mouse CD68 (APC-Cy7)	FA-11	Biolegend
anti-mouse CD69 (PE, PE-Dazzle594)	H1.2F3	Biolegend
anti-mouse CD8a (PerCP-Cy5.5, AF647)	53-6.7	Biolegend
anti-mouse CXCR5 (BV605)	L138D7	Biolegend
anti-mouse Foxp3 (unconjugated, AF647)	FJK-16s	Thermo Fisher
anti-mouse HSP90	C45G5	Cell Signaling
anti-mouse IFNγ	AN-18	Biolegend
anti-mouse IFNγ (biotin)	R4-6A2	Biolegend
anti-mouse IgG (H+L) (HRP)	polyclonal	Vector Laboratories
anti-mouse IgG-Fcγ fragement (AF647)	polyclonal	Jackson Immuno
anti-mouse IL17A	eBio17CK15A5	Thermo Fisher
anti-mouse IL17A (biotin)	eBio17B7	Thermo Fisher
anti-mouse IL2	JES6-1A12	Biolegend
anti-mouse IL2 (biotin)	JES6-5H4	Biolegend
anti-mouse IL4	11B11	Biolegend
anti-mouse IL4 (biotin)	BVD6-24G2	Biolegend
anti-mouse NK-1.1 (BV510)	PK136	Biolegend
anti-mouse NRP1	D62C6	Cell Signaling
anti-mouse NRP2	D39A5	Cell Signaling
anti-mouse TruStain FcX	---	Biolegend
anti-phycoerythrin (A0911 [DNA])	PE001	Biolegend
Streptavidin (FITC)	polyclonal	Thermo Fisher

Supplemental Table 2. Primers used in these studies.

target	reverse	forward
NRP2-WT	5` CAG GTG ACT GGG GAT AGG GTA 3`	5` AGC TTT TGC CTC AGG ACC CA 3`
NRP2lox	5` CCT GAC TAC TCC CAG TCA TAG 3`	
NRP2-WT	5` TGA AAT CAG CAA AGA GGG AGC 3`	5` GGG AAA CCC TCG TGA TGT TGT 3`
$NRP2^{KO}$	5` ATC TTC CAT CAC GTC GAA CTC 3`	
CD4-WT	5` TAT GCT CTA AGG ACA AGA ATT GAC A 3`	5` GTT CTT TGT ATA TAT TGA ATG TTA GCC 3`
CD4-cre	5` CTT TGC AGA GGG CTA ACA GC 3`	
Foxp3-WT	5` GTG TGA CTG CAT GAC TAA CTT TGA 3`	5` CCC TTG ACC TCA AAA CCA AG 3`
Foxp3-cre		5` TGG CTG GAC CAA TGT GAA C 3`
