# Supplementary material for: Neuropilin-2 functions as a coinhibitory receptor to regulate antigen-induced inflammation and allograft rejection
Source: J Clin Invest. 2025 Jul 1;135(13):e172218. doi: 10.1172/JCI172218 (PMC12208552; doi:10.1172/JCI172218)

Fully unedited blot for Supplemental Figure 5E – NRP2

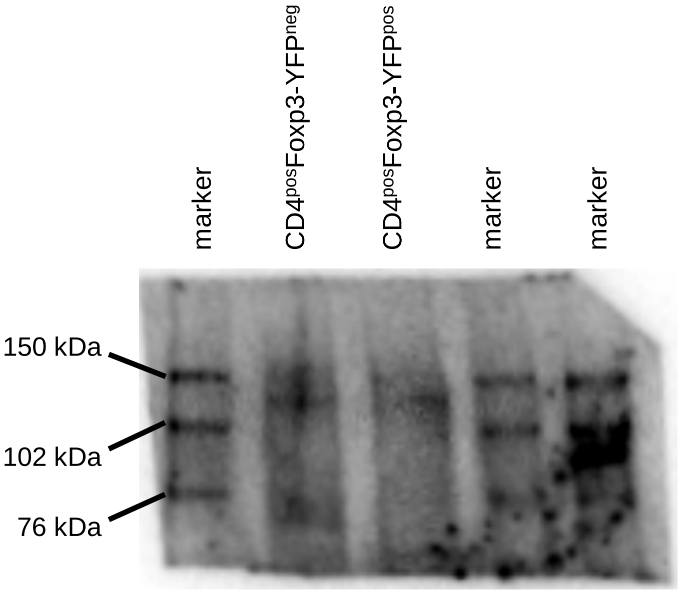

Chemiluminescence only

Fully unedited blot for Supplemental Figure 5E – NRP1

Chemiluminescence only

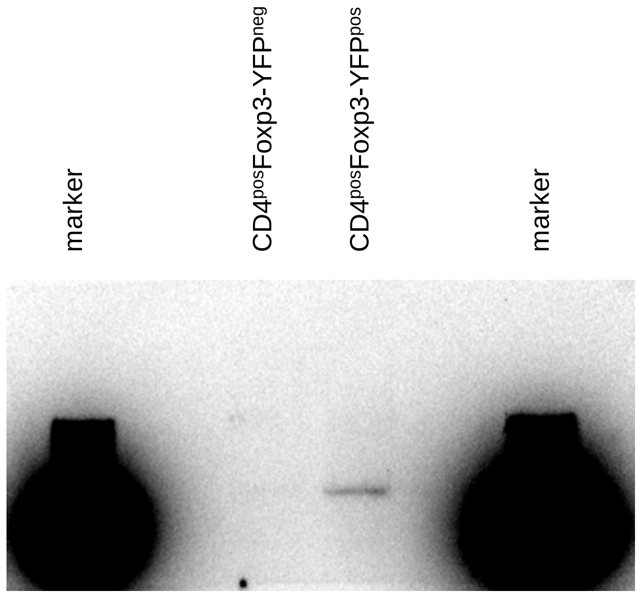

Chemiluminescence merged with picture

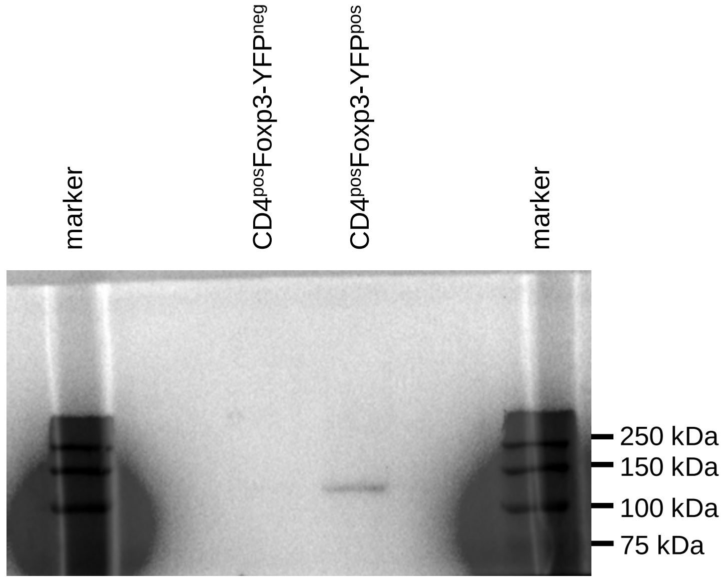

Fully unedited blot for Supplemental Figure 5E – Foxp3

Chemiluminescence only

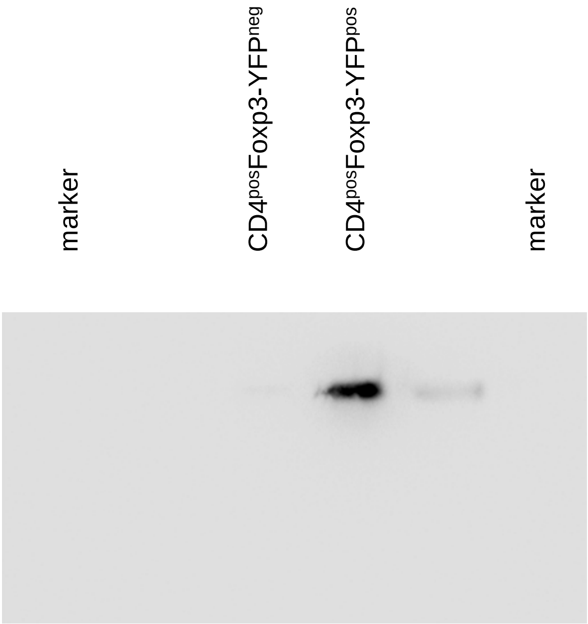

Chemiluminescence merged with picture

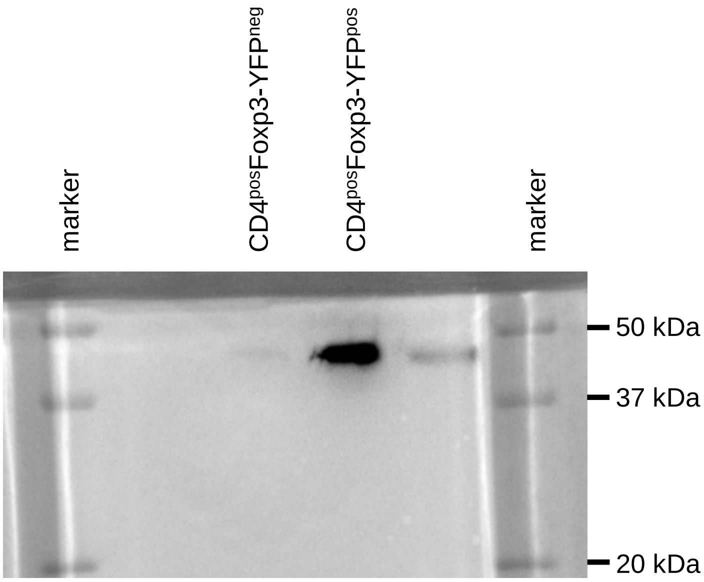

Fully unedited blot for Supplemental Figure 5E – HSP90

Chemiluminescence only

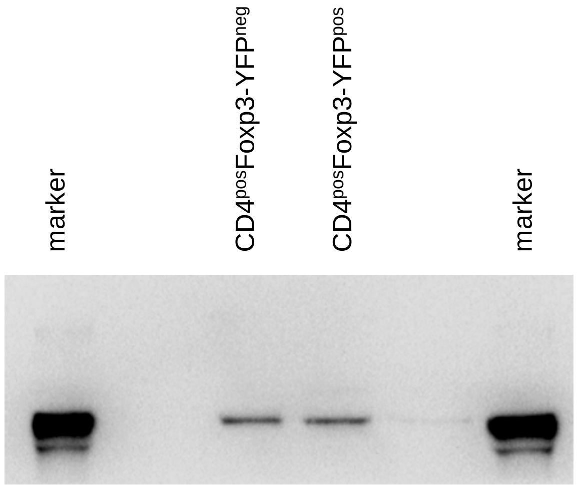

Chemiluminescence merged with picture

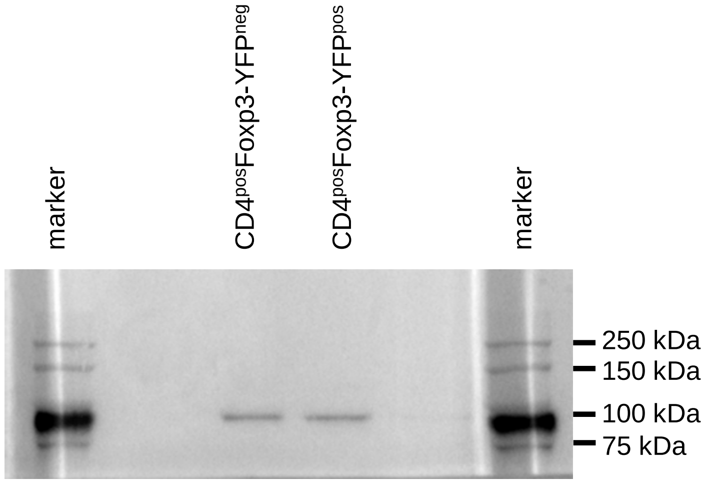

Fully unedited gel for Supplemental Figure 7A – top band

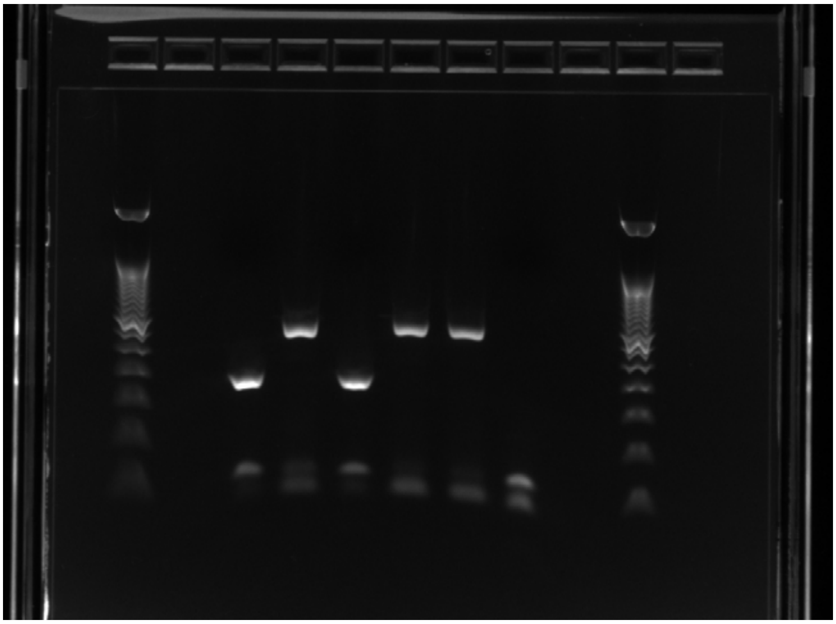

Fully unedited gel for Supplemental Figure 7A – 2nd band

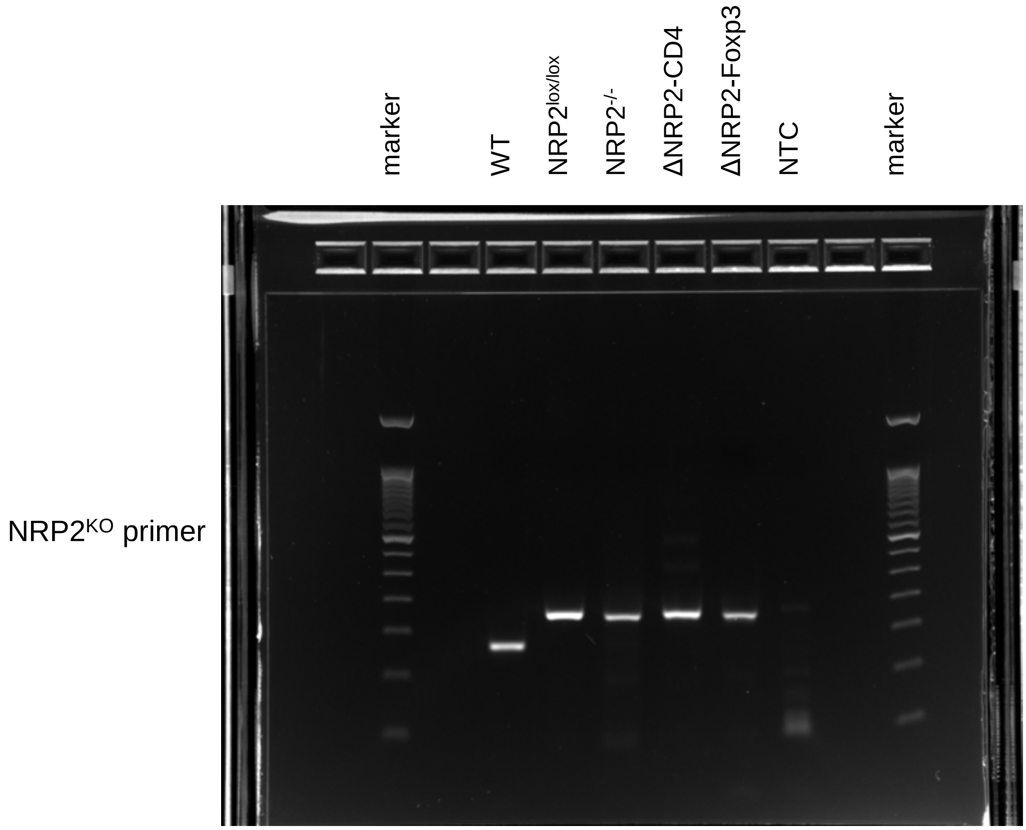

Fully unedited gel for Supplemental Figure 7A – 3rd band

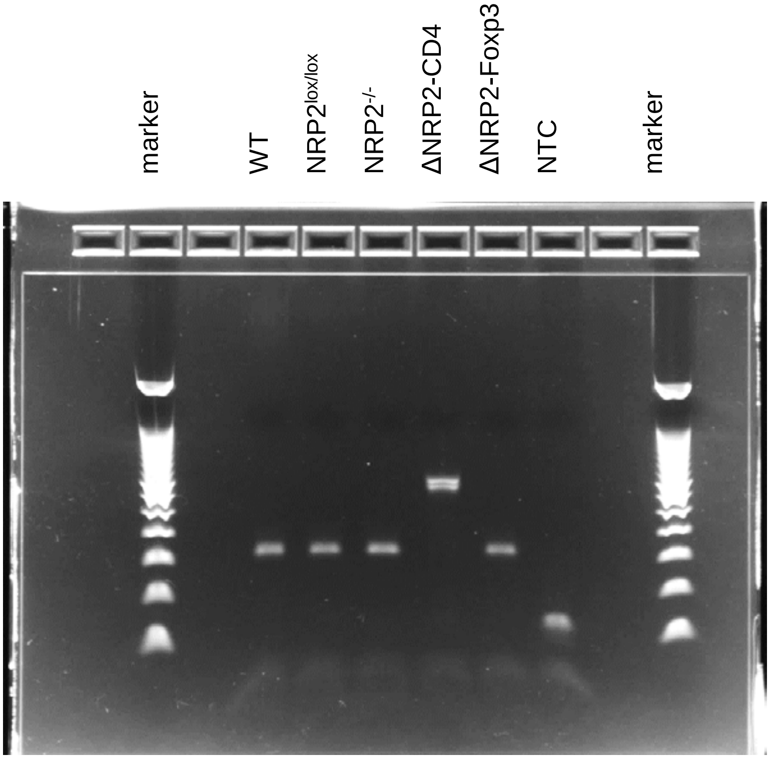

CD4-cre primer

Fully unedited gel for Supplemental Figure 7A – bottom band

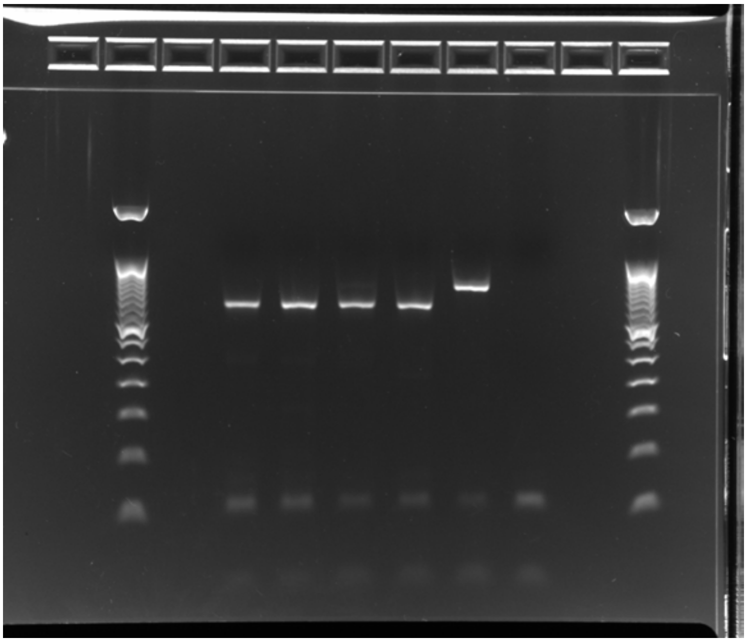

Supplement: Unedited blot and gel images [file jci-135-172218-s094.pdf]
